# Valores Ecocardiográficos de Referência para as Câmaras Cardíacas no Brasil: Um Estudo Multirregional e Multirracial

**DOI:** 10.36660/abc.20250628

**Published:** 2026-03-03

**Authors:** Ana Clara Tude Rodrigues, Roberto Magalhães Saraiva, Viviane T. Hotta, Gabriel Doreto Rodrigues, Fabiana Camelier de Assis Cardoso, Anderson da Costa Armstrong, Cláudia Gianini Monaco, Adelino Parro, José Luis de Castro e Silva Pretto, João Marcos Bemfica Barbosa Ferreira, Renata Santos Lopes Teixeira de Lima, Marcelo Salame, Salustiano Pereira de Araujo, Carlos Eduardo Suaide Silva, Cíntia Galhardo Tressino, Fabio Canellas Moreira, Ana Cristina Camarozano, Minna Moreira Dias Romano, Andre Luiz Cerqueira Almeida, Claudio Henrique Fischer, Alex dos Santos Felix, Clarissa Borguezan-Daros, Diogo Silva Piardi, Diego Cunha, Marcelo Dantas Tavares de Melo, Rafael Modesto, Silvio Henrique Barberato, Wercules Antonio Oliveira, Marcelo Luiz Campos Vieira, Samira Saady Morhy

**Affiliations:** 1 Hospital Israelita Albert Einstein São Paulo SP Brasil Hospital Israelita Albert Einstein, São Paulo, SP – Brasil; 2 Hospital das Clínicas Faculdade de Medicina Universidade de São Paulo São Paulo SP Brasil Instituto de Radiologia do Hospital das Clínicas da Faculdade de Medicina da Universidade de São Paulo (INRAD HCFMUSP), São Paulo, SP – Brasil; 3 Fundação Oswaldo Cruz Rio de Janeiro RJ Brasil Fundação Oswaldo Cruz, Rio de Janeiro, RJ – Brasil; 4 Fleury Medicina e Saúde Grupo Fleury São Paulo SP Brasil Fleury Medicina e Saúde, Grupo Fleury, São Paulo, SP – Brasil; 5 Hospital Universitário Maria Aparecida Pedrossian Universidade Federal de Mato Grosso do Sul Ebserh Campo Grande MS Brasil Hospital Universitário Maria Aparecida Pedrossian, Universidade Federal de Mato Grosso do Sul, Ebserh, Campo Grande, MS – Brasil; 6 Instituto D’Or de Pesquisa e Ensino Hospital CardioPulmonar Salvador BA Brasil Instituto D’Or de Pesquisa e Ensino (IDOR), Hospital CardioPulmonar Salvador, BA – Brasil; 7 Universidade Federal do Vale do São Francisco Petrolina PE Brasil Universidade Federal do Vale do São Francisco Curso de Medicina, Petrolina, PE – Brasil; 8 Instituto de Moléstias Cardiovasculares São José do Rio Preto SP Brasil Instituto de Moléstias Cardiovasculares (IMC), São José do Rio Preto, SP – Brasil; 9 Hospital São Vicente de Paulo Passo Fundo RS Brasil Hospital São Vicente de Paulo, Passo Fundo, RS – Brasil; 10 Universidade do Estado do Amazonas Manaus AM Brasil Universidade do Estado do Amazonas, Manaus, AM – Brasil; 11 Hospital Amecor Cuiabá MS Brasil Hospital Amecor, Cuiabá, MS – Brasil; 12 Jipamed Medicina Avançada Ji-Paraná RO Brasil Jipamed Medicina Avançada, Ji-Paraná, RO – Brasil; 13 Hospital Santa Genoveva Uberlândia MG Brasil Hospital Santa Genoveva, Uberlândia, MG – Brasil; 14 Diagnósticos da América SA Barueri SP Brasil Diagnósticos da América SA, Barueri, SP – Brasil; 15 Instituto Dante Pazzanese de Cardiologia Ecocardiografia São Paulo SP Brasil Instituto Dante Pazzanese de Cardiologia Ecocardiografia, São Paulo, SP – Brasil; 16 Santa Casa de Misericórdia de Porto Alegre Porto Alegre RS Brasil Santa Casa de Misericórdia de Porto Alegre, Porto Alegre, RS – Brasil; 17 Hospital de Clínicas Universidade Federal do Paraná Curitiba PR Brasil Hospital de Clínicas da Universidade Federal do Paraná, Curitiba, PR – Brasil; 18 Universidade de São Paulo Hospital das Clínicas Faculdade de Medicina de Ribeirão Preto Ribeirão Preto SP Brasil Universidade de São Paulo Hospital das Clínicas da Faculdade de Medicina de Ribeirão Preto, Ribeirão Preto, SP – Brasil; 19 Santa Casa de Misericórdia de Feira de Santana Cardiologia Feira de Santana BA Brasil Santa Casa de Misericórdia de Feira de Santana Cardiologia, Feira de Santana, BA – Brasil; 20 Instituto Nacional de Cardiologia Rio de Janeiro RJ Brasil Instituto Nacional de Cardiologia, Rio de Janeiro, RJ – Brasil; 21 Hospital São José Criciúma SC Brasil Hospital São José, Criciúma, SC – Brasil; 22 Universidade Federal da Paraíba João Pessoa PB Brasil Universidade Federal da Paraíba, João Pessoa, PB – Brasil; 23 Hospital Aliança Salvador BA Brasil Hospital Aliança, Salvador, BA – Brasil; 24 CardioEco Centro de Diagnóstico Cardiovascular Curitiba PR Brasil CardioEco Centro de Diagnóstico Cardiovascular, Curitiba, PR – Brasil; 25 Quanta Diagnóstico Ecocardiografia Curitiba PR Brasil Quanta Diagnóstico Ecocardiografia, Curitiba, PR – Brasil; 26 Hospital das Clínicas Faculdade de Medicina Universidade de São Paulo São Paulo SP Brasil Instituto do Coração do Hospital das Clínicas da Faculdade de Medicina da Universidade de São Paulo, São Paulo, SP – Brasil

**Keywords:** Ecocardiografia, Coração, Valores de Referência

## Abstract

**Fundamento:**

Valores de referência para a quantificação das câmaras cardíacas são essenciais para a tomada de decisão clínica. O Brasil é um país continental com grande diversidade racial e marcadas variações socioeconômicas e antropométricas regionais que podem não estar adequadamente representadas nos padrões ecocardiográficos internacionais.

**Objetivo:**

Estabelecer medidas ecocardiográficas de referência para adultos brasileiros saudáveis e investigar diferenças geográficas e relacionadas ao sexo nas principais regiões do país.

**Métodos:**

Neste estudo prospectivo e multicêntrico, ecocardiogramas transtorácicos foram realizados em 496 voluntários saudáveis (idade média de 41 ± 15 anos; 55% mulheres) provenientes de cinco regiões do Brasil. Foram analisadas as dimensões, os volumes, a massa, a fração de ejeção e o *strain* longitudinal global (SLG) do ventrículo esquerdo (VE), bem como os parâmetros do átrio esquerdo (AE), de acordo com as diretrizes atuais. As comparações foram realizadas por sexo (teste *t* de Student ou teste *U* de Mann-Whitney) e por região (análise de variância).

**Resultados:**

As dimensões das câmaras cardíacas e a massa do VE foram maiores nos homens, mesmo após indexação pela área de superfície corporal. Os volumes do AE também foram mais elevados nos homens, embora essas diferenças tenham sido atenuadas após a indexação. Os valores absolutos do SLG-VE e do SLG do ventrículo direito (VD) foram maiores nas mulheres, enquanto o *strain* de reservatório do AE (SRAE) foi semelhante entre os sexos. Em termos regionais, o Centro-Oeste apresentou volumes do VE significativamente menores, diferença principalmente observada nas mulheres, além de valores mais baixos do SLG-VE e do SLG-VD (p < 0,001). Em contraste, os volumes do AE foram menores na região Nordeste. Não foram observadas associações significativas entre raça autorreferida e parâmetros ecocardiográficos, exceto para o SLG-VD e o SRAE, que foram menores em participantes brancos.

**Conclusão:**

Este estudo estabelece valores ecocardiográficos de referência em uma coorte brasileira representativa em nível nacional, confirma as diferenças esperadas relacionadas ao sexo e identifica variações regionais previamente não reconhecidas nas dimensões das câmaras cardíacas. Esses achados reforçam a necessidade de padrões específicos para a população, a fim de garantir uma quantificação cardíaca mais precisa.

## Graphical abstract

**Figura Central:**
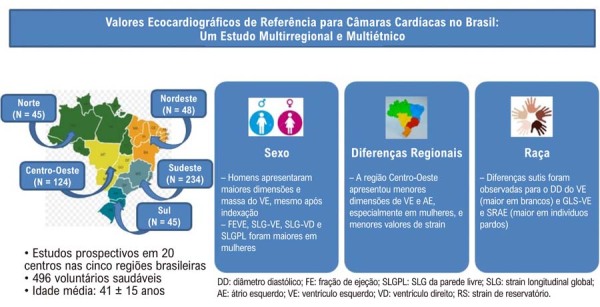
Valores Ecocardiográficos de Referência para as Câmaras Cardíacas no Brasil: Um Estudo Multirregional e Multirracial

## Introdução

A avaliação ecocardiográfica do tamanho das câmaras cardíacas, da massa do ventrículo esquerdo (VE) e da função sistólica é essencial para a caracterização do risco cardiovascular.^1 , 2^ As decisões terapêuticas são frequentemente orientadas por parâmetros de função sistólica, que fornecem informações valiosas para diagnóstico, tratamento e prognóstico em uma ampla variedade de condições. Além disso, a determinação precisa dos volumes cardíacos é fundamental para o acompanhamento de pacientes com insuficiência cardíaca, valvopatias e remodelamento cardíaco.^3^

Os valores de referência para medidas cardíacas normais podem refletir as características antropométricas, genéticas, socioeconômicas e raciais de populações específicas. O Brasil, um país de dimensões continentais, apresenta marcante diversidade econômica, racial e cultural. De acordo com o Censo de 2022, realizado pelo Instituto Brasileiro de Geografia e Estatística, a população brasileira é predominantemente composta por indivíduos pardos (45,31%), seguidos por brancos (43,5%), pretos (10,2%), indígenas (0,8%) e asiáticos (0,4%). Além disso, as cinco grandes regiões geográficas apresentam disparidades substanciais, cada uma com características próprias que podem influenciar desfechos em saúde e a expressão fenotípica.

A maioria dos estudos sobre valores ecocardiográficos de referência baseia-se em populações europeias^1^ ou norte-americanas,^2^ que podem não representar plenamente a população brasileira. Um estudo recente, de grande escala e multirracial, incluindo participantes de centros ao redor do mundo,^3^ demonstrou diferenças nos diâmetros e volumes do VE entre os países. Por exemplo, mulheres brasileiras apresentaram valores mais elevados de fração de ejeção (FE) do VE (FEVE) em comparação com aqueles relatados em coortes americanas, europeias e asiáticas.^3^

Em contraste, a maioria dos estudos conduzidos no Brasil foi limitada a coortes menores e de centro único,^4 , 5^ o que pode não capturar adequadamente a diversidade do país. Portanto, o objetivo deste estudo transversal foi estabelecer valores de referência para parâmetros ecocardiográficos em uma coorte robusta de indivíduos provenientes das cinco principais regiões brasileiras, além de comparar os achados de acordo com sexo e raça entre as regiões.

## Métodos

### Participantes e Cenário do Estudo

Voluntários saudáveis com idade > 18 anos, de ambos os sexos, foram prospectivamente incluídos. Os participantes foram recrutados entre funcionários de hospitais, familiares de funcionários e indivíduos submetidos a exames ecocardiográficos para avaliação de saúde ou *check-up* para fins de seguro ou plano de saúde.

Os critérios de exclusão incluíram qualquer condição cardiovascular ou sistêmica conhecida que pudesse influenciar as medidas cardíacas, como doença arterial coronariana, diabetes, hipertensão, arritmias, cardiopatias congênitas, doenças hepáticas ou pulmonares, distúrbios da tireoide ou renais e doenças reumáticas. Outros critérios de exclusão foram o uso de medicações cardiovasculares, índice de massa corporal (IMC) > 30 kg/m^2^, atletas (definidos como atletas profissionais ou indivíduos que praticam atividade esportiva regular por > 5 horas/semana), gestação, janelas ecocardiográficas subótimas, doença valvar significativa (regurgitação ou estenose maior que leve, detectada durante o exame) e FEVE < 50%.

A raça autorreferida foi classificada como parda, branca, preta, asiática ou indígena. Os participantes foram recrutados em 20 laboratórios de ecocardiografia distribuídos nas cinco principais regiões do Brasil: nove centros no Sudeste, quatro no Sul, três no Nordeste, dois no Centro-Oeste e dois no Norte ( Figura Central ).

### Medidas ecocardiográficas

A ecocardiografia transtorácica padrão foi realizada com equipamentos comercialmente disponíveis com capacidade de imagem harmônica. Os pacientes foram monitorados continuamente com eletrocardiografia (ECG), em posição de decúbito lateral esquerdo. Os ajustes de ganho e profundidade foram realizados para otimizar a qualidade da imagem.

Imagens bidimensionais foram adquiridas nas janelas paraesternais nos eixos longo e curto, bem como nas projeções apicais de quatro, três e duas câmaras. Os diâmetros diastólico final (DF) e sistólico final (SF) do VE foram medidos na janela paraesternal eixo longo para cálculo da massa do VE, juntamente com a espessura do septo interventricular e da parede posterior em DF, de acordo com as diretrizes da *American Society of Echocardiography* (ASE).

As projeções apicais de quatro e duas câmaras foram utilizadas para calcular os volumes DF e SF do VE, o volume sistólico (VS) e a FE pelo método de Simpson biplano modificado. Imagens obtidas nas projeções apicais de quatro, três e duas câmaras, com taxa de quadros > 50 quadros por segundo, foram utilizadas para a análise do *strain* longitudinal global (SLG) do VE.

Imagens da projeção apical de quatro câmaras focada no ventrículo direito (VD) foram obtidas para análise do SLG do VD e do SLG da parede livre (SLGPL). Os volumes do átrio esquerdo (AE) foram medidos nas projeções apicais de duas e quatro câmaras pelo método de Simpson, e o *strain* de reservatório do AE (SRAE) foi obtido a partir da projeção apical padrão de quatro câmaras. Os valores de *strain* foram analisados com base em sua magnitude absoluta, independentemente da representação negativa. Também foi realizada a medida linear do diâmetro anteroposterior do AE na janela paraesternal eixo longo, de acordo com as diretrizes da ASE.^6^

Os diâmetros e a massa do VE, bem como os volumes do VE e do AE, foram indexados à área de superfície corporal (ASC) pela fórmula de Du Bois.^7^ O Doppler colorido foi utilizado para avaliação de todas as valvas cardíacas. Doppler contínuo e pulsado foram obtidos nas valvas pulmonar, tricúspide, mitral e aórtica, nas janelas paraesternais e apicais, para avaliação do fluxo intracardíaco.

As imagens foram armazenadas no formato *digital imaging and communications in medicine* (DICOM) em DVDs e posteriormente carregadas em uma plataforma segura baseada na web para armazenamento e gerenciamento de dados de pesquisa, o Research Electronic Data Capture,^8^ juntamente com as informações clínicas para análise offline.

### Análise das Medidas

Todas as análises e medições das imagens foram realizadas *offline* , utilizando uma plataforma independente de fornecedor, capaz de armazenar, visualizar e analisar imagens ecocardiográficas. A análise de *strain* foi realizada *offline* com o *software* AutoStrain^®^, após confirmação da localização dos marcadores de início e término da diástole final (Ultrasound Workspace, Philips-Tomtec).

As análises das imagens foram realizadas por quatro pesquisadores de laboratórios centrais de ecocardiografia em São Paulo, Rio de Janeiro e Bahia.

### Considerações éticas

O estudo foi aprovado pelo Comitê de Ética do hospital coordenador (número de protocolo 17275419.3.1001.0071) e pelos comitês de ética de todos os centros participantes. Todos os participantes forneceram consentimento informado por escrito antes da inclusão. O estudo foi conduzido de acordo com os princípios éticos da Declaração de Helsinque e com a Resolução nº 466/2012 do Conselho Nacional de Saúde.

### Análise estatística

As medidas foram expressas como média e desvio padrão (DP) para variáveis com distribuição normal, ou como mediana e intervalo interquartil para variáveis com distribuição assimétrica. As variáveis contínuas também foram apresentadas como média ± DP. A distribuição das variáveis quantitativas foi avaliada pelo teste de Shapiro-Wilk. As variáveis categóricas foram expressas em porcentagens.

As comparações entre grupos foram realizadas por meio do teste *t* de Student não pareado ou do teste *U* de Mann-Whitney para variáveis contínuas, e do teste do qui-quadrado para variáveis categóricas. A análise de variância (ANOVA) ou o teste de Kruskal-Wallis foram utilizados para comparar diferenças entre subgrupos. O teste de Duncan foi aplicado como análise pós-hoc após a ANOVA, e o procedimento de Benjamini-Hochberg foi utilizado após os testes de Kruskal-Wallis. A significância estatística foi definida como p < 0,05.

**Figura 1:**
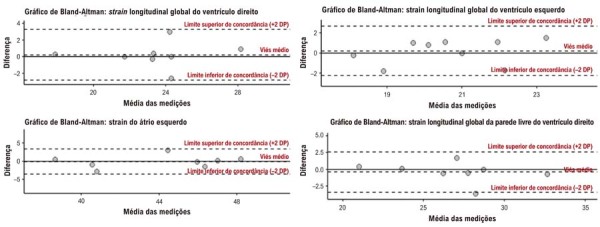
– Gráficos de Bland-Altman para a variabilidade intraobservador das medidas de strain. DP: desvio padrão.

**Figura 2:**
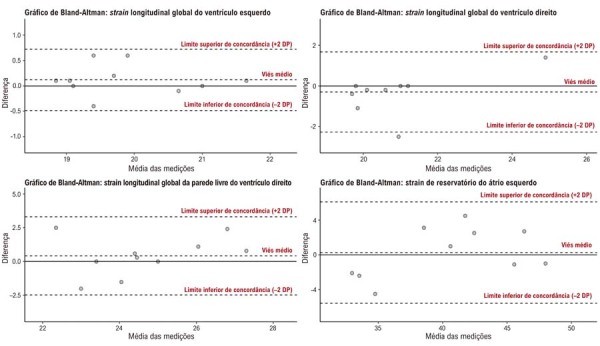
– Gráficos de Bland-Altman para a variabilidade interobservador nos parâmetros de strain. DP: desvio padrão.

A variabilidade intraobservador e interobservador foi avaliada por meio do coeficiente de correlação intraclasse em 10 pacientes e por gráficos de Bland-Altman realizados 30 dias após as medições iniciais ( Figura 1 ; Figura 2 ). A análise dos dados foi conduzida utilizando o software R, versão 4.1.1.

## Resultados

### Características da Amostra

A coleta de dados teve início em 2021, após o declínio da pandemia de covid-19. Um total de 593 indivíduos foi inicialmente incluído. Desses, 97 participantes foram excluídos pelos seguintes motivos: armazenamento inadequado das imagens (n = 75), ausência de registros de ECG (n = 10), IMC > 30 kg/m^2^ (n = 5), ausência de consentimento informado (n = 4), achados ecocardiográficos anormais (n = 2; um caso de prolapso da valva mitral e um de disfunção sistólica do VE) e idade < 18 anos (n = 1). Assim, 496 participantes foram incluídos na análise final.

Os participantes foram recrutados em 20 laboratórios de ecocardiografia distribuídos nas cinco principais regiões brasileiras; dois centros contribuíram apenas como locais de leitura. A maioria dos participantes (n = 234) era da região Sudeste, a mais populosa do Brasil. A amostra restante incluiu 124 indivíduos do Centro-Oeste, 48 do Nordeste, 45 do Sul e 45 do Norte ( Figura Central ).

A análise da raça autorreferida mostrou que a maioria dos participantes se identificou como branca (56%), seguida por pardos (31%) e pretos (11%). Uma proporção menor se identificou como asiática (2%), e nenhum participante relatou ancestralidade indígena. A distribuição da raça autorreferida variou significativamente entre as regiões. Indivíduos pardos representaram 63% e 53% dos participantes no Nordeste e no Norte, respectivamente, enquanto no Sul 84% se identificaram como brancos. A Tabela 1 apresenta a distribuição da raça autorreferida por região.

**Tabela 1:** – Características raciais da população brasileira de acordo com a região

Raça	Total (n = 496)	SE (n = 234)	CO (n = 124)	NE (n = 48)	S (n = 45)	N (n = 45)
Branca, n (%)	277 (56,3)	130 (55,6)	76 (61,3)	14 (29,2)	38 (84,4)	19 (42,2)
Parda, n (%)	157 (31,3)	61 (26,0)	35 (28,2)	30 (62,5)	7 (15,6)	24 (53,3)
Preta, n (%)	53 (10,6)	34 (14,5)	13 (10,5)	4 (8,3)	0 (0)	2 (4,4)
Asiática, n (%)	9 (1,8)	9 (3,9)	0 (0)	0 (0)	0 (0)	0 (0)

CO: Centro-Oeste; N: Norte; NE: Nordeste; S: Sul; SE: Sudeste.

A população do estudo incluiu uma proporção ligeiramente maior de mulheres (55%). A idade média foi de 41 ± 15 anos (variação: 18-100 anos), sem diferença significativa entre homens e mulheres (p = 0,30). Como esperado, as mulheres apresentaram medidas antropométricas significativamente menores que os homens, incluindo peso, altura, ASC e IMC. As mulheres também apresentaram frequência cardíaca de repouso mais elevada (p < 0,001). Um resumo detalhado das características demográficas da coorte geral e estratificadas por sexo é apresentado na Tabela 2 .

**Tabela 2:** – Dados demográficos e frequência cardíaca de todos os indivíduos (homens e mulheres)

Variável	Total (n = 496)	Homens (n = 224)	Mulheres (n = 272)	Valor p
**Idade (anos)**				0,312
Média [DP] (IC 95%)	41,4 [15,0] (40,0-42,7)	40,5 [14,7] (38,6-42,4)	42,0 [15,2] (40,2-43,9)	
Mediana (IC 95%)	39 (37-41)	38 (35-40)	40 (38-43)	
**Peso (kg)**				< 0,001
Média [DP] (IC 95%)	71,0 [11,5] (70,0-72,0)	77,9 [10,0] (76,6-79,2)	65,3 [9,4] (64,2-66,5)	
Mediana (IC 95%)	70 (70-72)	77 (72-84)	64 (63-66)	
**Altura (cm)**				< 0,001
Média [DP] (IC 95%)	167,7 [9,2] (166,9-168,5)	174,3 [7,3] (173,3-175,2)	162,3 [6,7] (161,5-163,1)	
Mediana (IC 95%)	168 (166-169)	174 (172-175)	162 (161-164)	
**ASC (m** ^**2**^ **)**				< 0,001
Média [DP] (IC 95%)	1,80 [0,18] (1,78-1,82)	1,93 [0,15] (1,91-1,95)	1,70 [0,14] (1,68-1,71)	
Mediana (IC 95%)	1,79 (1,70-1,82)	1,91 (1,89-1,93)	1,69 (1,67-1,71)	
**IMC (kg/m** ^**2**^ **)**				< 0,001
Média [DP] (IC 95%)	25,1 [2,8] (24,9-25,4)	25,6 [2,5] (25,3-25,9)	24,7 [2,9] (24,4-25,1)	
Mediana (IC 95%)	25,2 (24,8-25,5)	25,6 (25,3-25,9)	24,5 (24,0-25,0)	
**Frequência cardíaca (bpm)**				< 0,001
Média [DP] (IC 95%)	69,3 [10,4] (68,5-70,3)	67,3 [10,2] (66,0-68,7)	71,1 [10,3] (69,8-72,3)	
Mediana (IC 95%)	69 (68-70)	66 (60-74)	71 (70-73)	

IC: intervalo de confiança; IMC: índice de massa corporal; ASC: área de superfície corporal; DP: desvio padrão.

### Parâmetros ecocardiográficos

Todas as dimensões, volumes e massa do VE foram maiores nos homens, e essas diferenças relacionadas ao sexo permaneceram significativas mesmo após a indexação pela ASC. Tanto o diâmetro anteroposterior do AE quanto o volume do AE foram maiores nos homens, embora os volumes do AE indexados tenham sido semelhantes entre os sexos.

Em relação à função sistólica do VE, as mulheres apresentaram valores ligeiramente mais altos de FEVE (63,1 ± 4,6% vs. 61,4 ± 4,3% para mulheres e homens, respectivamente; Tabela Suplementar 3). A maioria dos parâmetros de deformação miocárdica demonstrou diferenças marcantes relacionadas ao sexo. As mulheres apresentaram valores significativamente mais altos de SLG-VE, SLG-VD e SLGPL em comparação aos homens. Embora o SRAE também tenha apresentado tendência a valores mais altos nas mulheres, a diferença não atingiu significância estatística (p = 0,089).

### Diferenças regionais nas características demográficas e nos parâmetros do ventrículo esquerdo

A distribuição por sexo foi semelhante em todas as regiões brasileiras. No entanto, os indivíduos do Nordeste eram ligeiramente mais jovens do que aqueles do Sudeste e do Centro-Oeste (p = 0,003). A ASC foi ligeiramente maior no Sudeste em comparação com o Sul (p = 0,027).

Observou-se variação regional significativa nas medidas do VE. Os volumes DF (p < 0,001) e SF (p = 0,017), bem como os diâmetros (p < 0,05), foram menores no Centro-Oeste, particularmente em comparação com o Norte, mesmo após a indexação pela ASC. O volume sistólico também foi menor no Centro-Oeste (p < 0,001), e essa diferença permaneceu significativa após a indexação (p < 0,001). Em contraste, a FEVE não diferiu significativamente entre as regiões.

A massa do VE, o índice de massa do VE e a espessura do septo interventricular foram semelhantes entre as regiões; no entanto, a espessura da parede posterior foi significativamente menor no Nordeste (7,7 ± 0,9 mm) em comparação com o Norte (8,2 ± 1,1 mm; p = 0,006).

Também foi observada variação regional nos parâmetros de deformação miocárdica. O SLG-VE diferiu significativamente entre as regiões (p < 0,001), com o Centro-Oeste apresentando consistentemente os menores valores nas comparações pós-hoc. Um padrão semelhante foi observado para o SLG-VD e o SLGPL-VD (ambos p < 0,001), novamente com os menores valores no Centro-Oeste. Em contraste, o SRAE também variou significativamente entre as regiões (p = 0,043), com os maiores valores observados no Nordeste (Tabela Suplementar 4).

### Diferenças relacionadas ao sexo entre as regiões

Entre as regiões, os homens apresentaram poucas diferenças nos parâmetros ecocardiográficos; o volume DF do VE e o volume sistólico foram menores no Centro-Oeste. Entre as mulheres, entretanto, foram observadas diferenças regionais significativas tanto nas variáveis demográficas quanto nas ecocardiográficas.

As mulheres do Nordeste eram mais jovens do que aquelas das outras regiões. Do ponto de vista ecocardiográfico, o Centro-Oeste apresentou os menores diâmetros diastólico e sistólico finais do VE (p < 0,05), bem como os menores volumes DF e SF do VE (p < 0,001), enquanto o Norte apresentou os maiores volumes.

O volume do AE (p = 0,002) e o volume do AE indexado (p = 0,003) foram menores nas mulheres do Nordeste. Embora a massa do VE e o índice de massa não tenham diferido significativamente entre as mulheres, alguma variação regional foi observada na espessura miocárdica. Especificamente, a espessura da parede posterior (p = 0,006) e a espessura do septo interventricular (p = 0,005) foram significativamente menores nas mulheres do Norte, padrão observado apenas no subgrupo feminino.

Para os parâmetros de *strain* , foram observadas diferenças regionais em ambos os sexos. Entre os homens, o SLG-VE e o SLG-VD variaram significativamente entre as regiões, com o menor SLG-VE observado no Centro-Oeste (19,3 ± 1,2%) e o maior no Sul (21,8 ± 2,3%). O SLG-VD também foi menor no Centro-Oeste em comparação com as demais regiões.

Entre as mulheres, o SLG-VE, o SLG-VD, o SLGPL e o SRAE apresentaram diferenças regionais significativas. De forma semelhante aos homens, o Centro-Oeste apresentou os menores valores de SLG-VE (20,4 ± 1,8%) e SLG-VD (21,8 ± 2,2%) nas mulheres. Notavelmente, as mulheres do Sul apresentaram os maiores valores de SLG-VE (23,3 ± 2,3%) e de SRAE (43,7 ± 5,5%). As análises pós-hoc confirmaram que o Centro-Oeste diferiu consistentemente de várias outras regiões em ambos os sexos, particularmente em relação ao *strain* do VE e do VD (Tabela Suplementar 5).

### Diferenças de Acordo Com a Raça Autorreferida

Como a raça tem sido associada a variações nas medidas ecocardiográficas,^4^ avaliamos possíveis diferenças entre os dois maiores subgrupos raciais: pardos (n = 157) e brancos (n = 277). O diâmetro DF do VE indexado foi significativamente maior nos participantes brancos. Em contraste, o SLG-VD (23,1 ± 2,8% vs. 22,5 ± 2,5%; p = 0,036) e o SRAE (41,5 ± 5,8% vs. 40,3 ± 5,9%; p = 0,039) foram significativamente maiores no grupo pardo.

Não foram encontradas diferenças significativas para o SLG-VE ou para o SLGPL. Entre as variáveis demográficas, apenas a idade diferiu significativamente, sendo ligeiramente maior entre os participantes brancos (p = 0,025); todas as demais características foram comparáveis entre os grupos (Tabela Suplementar 6).

## Discussão

### População

Este é o primeiro estudo prospectivo e multicêntrico a fornecer parâmetros de referência ecocardiográficos bidimensionais e de deformação miocárdica para a população adulta brasileira, incluindo participantes de todas as cinco principais regiões do país, com análises estratificadas por sexo e raça autorreferida. De acordo com o Censo de 2022,^5^ a maior parte da população brasileira é composta por indivíduos pardos. No entanto, em nossa amostra, a maioria dos participantes se autodeclarou branca (57%), seguida por pardos (31%) e pretos (10%), com uma pequena proporção identificando-se como asiática (2%).

Reconhecemos que muitos indivíduos que se autodeclaram brancos podem apresentar características compatíveis com ancestralidade mista. A população brasileira reflete uma complexa miscigenação de ancestrais, principalmente portugueses, africanos e indígenas, posteriormente complementada por migrações europeias e japonesas, resultando em um histórico genealógico altamente heterogêneo. Assim, os indivíduos podem ter múltiplas origens ancestrais, embora isso nem sempre seja refletido na raça autorreferida.

Notavelmente, uma maior proporção de participantes foi recrutada em serviços privados de saúde, em vez de instituições públicas. Como indivíduos pardos e pretos no Brasil, em geral, têm menor acesso à saúde privada do que indivíduos brancos, esse padrão de recrutamento pode ter introduzido viés de seleção.^6^ Em consonância com os dados mais recentes do censo, a região Sul apresentou maior proporção de indivíduos brancos, enquanto as regiões Nordeste e Norte foram predominantemente compostas por indivíduos pardos.

Quanto à distribuição por sexo, nosso estudo incluiu uma proporção ligeiramente maior de mulheres (55%), refletindo de perto a população brasileira, na qual as mulheres correspondem a aproximadamente 51%. A maioria dos participantes foi recrutada na região Sudeste, que abriga as maiores e mais populosas cidades do país e representa mais de 40% da população brasileira, evidenciando as marcantes disparidades geográficas do país.

Incluímos indivíduos em uma ampla faixa etária (18-100 anos), com idade média de 41 ± 15 anos, ligeiramente superior à média nacional reportada no censo mais recente (35 anos). Em razão dessas características, acreditamos que nossa amostra possa ser amplamente representativa da população brasileira. No entanto, em comparação com alguns estudos internacionais, nossa coorte é mais jovem, com idade média de 45,8 anos no estudo *Normal Reference Ranges for Echocardiography* ^7^ e 57 anos em uma coorte longitudinal norueguesa.^8^

### Diferenças entre os sexos

Em nossa coorte, as dimensões das câmaras do VE foram maiores nos homens, em concordância com relatos anteriores, e essas diferenças persistiram mesmo após a indexação pela ASC. Os valores médios foram menores do que aqueles relatados em outros estudos,^8^ o que provavelmente reflete diferenças demográficas, incluindo menor altura, peso e ASC, fatores conhecidos por influenciar a estrutura cardíaca.

Os volumes do AE também foram significativamente maiores nos homens; entretanto, essas diferenças deixaram de ser significativas após a indexação pela ASC. Notavelmente, mesmo após a indexação, os volumes do AE em nossa população permaneceram menores do que aqueles relatados em coortes europeias (25,9 ± 6,3 mL/m^2^ em homens e 25,6 ± 6,0 mL/m^2^ em mulheres).

Em relação à FEVE, nossa análise demonstrou valores mais elevados nas mulheres em comparação aos homens, em concordância com estudos prévios e possivelmente relacionados a diferenças entre os sexos no volume sistólico ou na pós-carga.^9^ Nossos achados são consistentes com os do estudo *World Alliance Societies of Echocardiography* , embora a coorte brasileira desse estudo tenha sido derivada de um único centro. Estudos anteriores realizados no Brasil também propuseram valores de referência para a população,^10 , 11^ mas igualmente se trataram de investigações unicêntricas envolvendo indivíduos de apenas uma região.

Avanços tecnológicos em imagem e a melhora da resolução espacial têm ampliado a visualização das estruturas cardíacas, permitindo medições bidimensionais mais precisas. Em relação à deformação miocárdica, nossos achados confirmam que os parâmetros de *strain* são influenciados pelo sexo, com mulheres apresentando consistentemente valores mais elevados (mais negativos) em ambos os ventrículos, corroborando diferenças relacionadas ao sexo previamente descritas.^12 - 14^

No Estudo Longitudinal de Saúde do Adulto (ELSA-Brasil),^15^ foram reportados valores mais baixos de SLG-VE, embora valores mais altos nas mulheres também tenham sido confirmados. Diferentemente do presente estudo, as imagens ecocardiográficas no ELSA-Brasil não foram adquiridas prospectivamente com o objetivo específico de análise de *strain* , e o *strain* do VE foi derivado apenas das projeções apicais de duas e quatro câmaras, sem inclusão da projeção de três câmaras. Essa diferença metodológica pode explicar os valores mais baixos observados.

O SLG-VE mais negativo observado nas mulheres, em comparação aos homens, está em linha com estudos anteriores que sugerem que as mulheres apresentam mecânica sistólica mais eficiente, possivelmente devido a diferenças na orientação das fibras miocárdicas, menor pós-carga e influências hormonais. Além disso, o SLG-VD e o SLGPL foram significativamente mais elevados nas mulheres, provavelmente refletindo diferenças entre os sexos na geometria do VD, no acoplamento ventrículo-vascular e nos efeitos hormonais. Níveis elevados de estradiol em mulheres em terapia hormonal correlacionam-se com maior FEVD e menor volume sistólico final, sustentando a influência benéfica do estrogênio na estrutura e função do VD.^16^

É importante destacar que o *strain* do VD tem emergido como um marcador mais sensível da função do VD do que parâmetros tradicionais, como o deslocamento sistólico do plano do anel tricúspide,^17^ e valores normais específicos por sexo devem ser considerados para evitar sub ou superestimação da disfunção do VD. Estudos experimentais também demonstraram que alterações no *strain* permitem detectar modificações cardíacas específicas por sexo em estágios mais precoces quando comparadas aos métodos ecocardiográficos convencionais.^18^

Esses achados reforçam a importância da estratificação por sexo em bancos de dados normativos ecocardiográficos, particularmente para parâmetros de deformação miocárdica, que têm sido cada vez mais incorporados às diretrizes clínicas para avaliação da função cardíaca. Além de ser mais sensível e reprodutível do que medidas tradicionais, como a FE,^19^ o SLG apresenta menor variabilidade inter e intraobservador, especialmente quando medido por *softwares* automatizados, como o AutoStrain.^20^

### Principais regiões geográficas

Embora o estudo tenha buscado obter uma amostragem representativa nas cinco regiões do Brasil, a maioria dos participantes foi recrutada no Sudeste, a área mais densamente povoada do país. Ainda assim, nossa análise identificou pequenas, porém estatisticamente significativas, diferenças regionais. Os diâmetros e volumes do VE foram menores na região Centro-Oeste. Esse achado pode estar parcialmente relacionado à composição racial dessa população, que inclui maior ancestralidade indígena, embora de forma menos pronunciada do que na região Norte.

Em relação aos volumes do AE, mulheres do Nordeste apresentaram valores menores, especialmente quando comparadas às do Sul, mesmo após ajuste pela ASC. Essa diferença pode refletir, em parte, a menor idade das participantes do Nordeste, uma vez que o volume do AE tende a aumentar com o envelhecimento devido à disfunção diastólica progressiva e ao remodelamento atrial. Considerando a relevância prognóstica dos volumes do AE, essas variações regionais merecem atenção.^21^ As espessuras do septo interventricular e da parede posterior também foram menores nas mulheres do Nordeste, provavelmente refletindo uma combinação de influências genéticas e raciais.

Quanto à deformação miocárdica, observou-se variação regional significativa no SLG-VE, SLG-VD, SLGPL e SRAE. O Centro-Oeste apresentou consistentemente valores mais baixos em todos os parâmetros, possivelmente relacionados às menores dimensões do VE e, consequentemente, a menores demandas volumétricas. Em contraste, participantes do Nordeste apresentaram valores mais elevados de *strain* , particularmente para SLG-VE e SLGPL, o que pode estar associado a diferenças antropométricas, níveis de atividade física ou fatores genéticos. Valores mais altos de SRAE no Nordeste também podem refletir melhor complacência atrial relacionada à menor idade desse grupo.

De modo geral, esses achados demonstram variabilidade geográfica relevante nos parâmetros de deformação miocárdica, especialmente entre indivíduos do Centro-Oeste, que consistentemente apresentaram valores mais baixos de *strain* do VE e do VD em ambos os sexos. As diferenças mais pronunciadas observadas nas mulheres, incluindo variações significativas no SLGPL e no SRAE, reforçam a importância de estabelecer valores de referência específicos por sexo e por região para a interpretação adequada dos parâmetros de *strain* em populações diversas.

### Raça autorreferida

Para qualquer população, os valores de referência normais são influenciados por características antropométricas e raciais.^7^ Evidências sugerem que diferenças raciais também podem afetar morbidade e mortalidade, particularmente na presença de desigualdades socioeconômicas.^22^ Os intervalos de referência em ecocardiografia variam de acordo com a raça,^4^ o que pode contribuir para diferenças no tamanho do VE e do AE.

Em nossa coorte, foram observadas diferenças sutis relacionadas à raça na função miocárdica subclínica. Indivíduos pardos apresentaram maior SLG-VD e valores mais elevados de SRAE. Essas variações podem refletir adaptações fisiológicas relacionadas a fatores genéticos, hemodinâmicos ou antropométricos. Embora tais diferenças possam estar parcialmente associadas ao remodelamento cardíaco relacionado à idade, sua relevância clínica parece limitada, uma vez que todos os valores permaneceram dentro das faixas de normalidade.

As dimensões cardíacas também estão intimamente relacionadas ao tamanho corporal e, neste estudo, a ASC foi semelhante entre os grupos. Um estudo brasileiro anterior de valores de referência não encontrou associação entre parâmetros ecocardiográficos e raça.^10^ Por fim, assim como no Censo brasileiro, a raça neste estudo foi baseada em autodeclaração. A identificação racial no Brasil baseia-se, em grande parte, no fenótipo, e não na linhagem familiar; portanto, o uso de coortes racialmente diversas é essencial para minimizar possíveis erros de classificação na interpretação ecocardiográfica.

### Limitações do estudo

Apesar do grande tamanho amostral, algumas regiões incluíram números relativamente pequenos de participantes, o que limita a capacidade de avaliar plenamente as diferenças regionais. Além disso, o número de participantes asiáticos foi reduzido, impedindo comparações significativas entre subgrupos raciais. Este estudo não incluiu indivíduos indígenas, que representam aproximadamente 0,83% da população brasileira (quase 2 milhões de pessoas). Pesquisas envolvendo comunidades indígenas no Brasil exigem autorização específica da Comissão Nacional de Ética em Pesquisa e frequentemente apresentam desafios logísticos relacionados à acessibilidade limitada. Portanto, nossos achados não podem ser generalizados para esse grupo.

Embora múltiplas plataformas de ultrassom tenham sido utilizadas nos diferentes centros participantes, todas as aquisições foram exportadas em formato DICOM e analisadas por meio de *software* independente de fabricante, reduzindo a variabilidade entre sistemas. Por fim, não foram realizadas análises por subgrupos etários, uma vez que apenas 52 participantes tinham mais de 65 anos, e foi difícil identificar indivíduos nessa faixa etária que atendessem a todos os critérios de inclusão (ausência de hipertensão, diabetes ou outras doenças cardiovasculares).

## Conclusão

Este é o primeiro estudo multicêntrico a fornecer valores ecocardiográficos de referência representativos em nível nacional para o Brasil. Além de confirmar diferenças relacionadas ao sexo, identificamos variabilidade regional previamente não reconhecida, ressaltando a importância de padrões ecocardiográficos específicos para a população.

## Material suplementar

Table 3
