# Errata

**DOI:** 10.36660/abc.20260248

**Published:** 2026-05-08

Edição de Fevereiro de 2026, vol. 123(2):e20250628

No Artigo Original “Valores Ecocardiográficos de Referência para as Câmaras Cardíacas no Brasil: Um Estudo Multirregional e Multirracial”, com número de DOI: https://doi.org/10.36660/abc.20250628, publicado no periódico Arquivos Brasileiros de Cardiologia, Arq Bras Cardiol. 2026; 123(2):e20250628, na página 1, incluir a instituição: Complexo Hospitalar Américas,27, Rio de Janeiro, RJ – Brasil. Alterar a numeração das afiliações do Dr. Alex dos Santos Felix de 15,20 para 14,20,27.

