# Supplementary material for: Valores Ecocardiográficos de Referência para as Câmaras Cardíacas no Brasil: Um Estudo Multirregional e Multirracial
Source: Arq Bras Cardiol. 2026 Mar 3;123(2):e20250628. [Article in Portuguese] doi: 10.36660/abc.20250628 (PMC13128182; doi:10.36660/abc.20250628)
Supplement: Table 3 [file 0066-782x-abc-123-2-e20250628-suppl01.pdf]

Table 3: Echocardiographic measurements of all individuals and subgroups according to sex.

	All (n= 496)<br>Mean [SD]<br>(2SD range)	Male (n=224)<br>Mean [SD]<br>(2SD range)	Female (n= 272)<br>Mean [SD]<br>(2SD range)	p-value
LA<br>(mm)	33.6 [3.3]<br>(27.0 - 40.1)	35.1 [3.2]<br>(28.8 - 41.2)	32.4 [2.9]<br>(26.5 - 38.3)	<0.001
LVEDD<br>(mm)	45.6 [3.9]<br>(38.0 - 53.2)	47.5 [3.5]<br>(40.5 - 54.5)	44.1 [3.4]<br>(37.4 - 50.7)	<0.001
LVESD<br>(mm)	29.9 [3.0]<br>(24.0 - 35.8)	31.9 [3.0]<br>(25.3 - 37.1)	28.9 [2.6]<br>(23.8 – 34.0)	<0.001
LVEDD index<br>(mm/m^2)	25.5 [2.3]<br>(20.9 - 30.2)	24.8 [2.1]<br>(20.6 - 28.9)	26.1 [2.4]<br>(21.4 - 30.8)	<0.001
LVESD index<br>(mm/m^2)	16.7 [1.7]<br>(13.2 - 20.2)	16.2 [1.6]<br>(13.0 - 19.5)	17.1 [1.8]<br>(13.6 - 20.5)	<0.001
SWt<br>(mm)	8.7 [1.2]<br>(6.4 – 11.0)	9.2 [1.2]<br>(7.0 - 11.5)	8.3 [1.1]<br>(6.3 - 10.4)	<0.001
PWt<br>(mm)	8.1 [1.1]<br>(6.0 - 10.2)	8.6 [1.1]<br>(6.5 - 10.6)	7.7 [0.9]<br>(5.9 - 9.6)	<0.001
LV Mass<br>(g)	128.8 [32.2]<br>(65.7 – 192.0)	147.2[32.3]<br>(83.8 - 210.6)	113.7 [22.9]<br>(68.8 - 158.6)	<0.001
LV Mass index<br>(g/m^2)	71.2 [14.7]<br>(42.4 - 100.2)	76.4 [15.6]<br>(45.8 - 106.9)	67.1 [12.6]<br>(42.4 - 91.7)	<0.001
LA Vol<br>(ml)	43.3 [10.0]<br>(23.6 – 63.0)	46.4 [10.4]<br>(26.0 - 66.8)	40.7 [8.9]<br>(23.2 - 58.3)	<0.001
LA Vol index<br>(ml/m^2)	24.1 [5.0]<br>(14.2 - 33.9)	24.1 [5.1]<br>(14.2 – 34.0)	24.0 [4.9]<br>(14.3 - 33.8)	0.691
LVED Vol<br>(ml)	87.8 [20.7]<br>(47.1 - 128.5)	100.1 [19.8]<br>(61.3 - 138.8)	77.7 [15.4]<br>(47.5 - 107.7)	<0.001
LVES Vol<br>(ml)	33.2 [9.5]<br>(14.5 - 51.9)	38.7 [9.4]<br>(20.3 - 57.1)	28.6 [6.9]<br>(15.1 - 42.2)	<0.001
LVED Vol index<br>(ml/m^2)	48.6 [9.7]<br>(29.8 - 67.4)	51.9 [9.5]<br>(33.3 - 70.6)	45.8 [8.7]<br>(28.8 - 62.8)	<0.001
LVES Vol index<br>(ml/m^2)	18.3 [4.5]<br>(9.6 - 27.1)	20.1 [4.4]<br>(11.3 - 28.8)	16.9 [3.9]<br>(9.2 - 24.6)	<0.001
Stroke Vol<br>(ml)	54.2 [12.9]<br>(28.9 - 79.4)	60.6 [12.6]<br>(35.8 - 85.4)	48.8 [10.4]<br>(28.4 - 69.1)	<0.001
Stroke Vol index<br>(ml/m^2)	30.0 [6.2]<br>(17.8 - 42.3)	31.5 [6.3]<br>(19.1 - 43.9)	28.8 [5.9]<br>(17.2 - 40.4)	<0.001
LVEF<br>(%)	62.3 [4.7]<br>(53.4 - 71.3)	61.4 [4.3]<br>(53.0 - 69.9)	63.1 [4.6]<br>(54.0 - 72.2)	<0.001
LV GLS (%)	21.1 [2.6]<br>(16.1 - 26.2)	20.6 [1.8]<br>(17.0 - 24.2)	21.6 [2.9]<br>(15.7 - 27.4)	<0.001
RV GLS (%)	22.7 [2.6]<br>(17.5 - 27.9)	22.2 [2.4]<br>(17.4 - 27.1)	23.1 [2.7]<br>(17.7 - 28.5)	<0.001
RVFWLS (%)	26.9 [3.6]<br>(19.9 - 34.1)	26.4 [3.4]<br>(19.7 - 33.2)	27.4 [3.6]<br>(20.2 - 34.7)	0.002
LARS (%)	40.8 [5.8]<br>(29.4 - 52.3)	40.3 [5.8]<br>(28.9 - 51.7)	41.2 [5.8]<br>(29.8 - 52.6)	0.089

LA = left atrium; LV = left ventricular; ED = end-diastolic; D= diameter; ES = end-systolic; SWt = septal wall thickness; PWt = posterior wall thickness; Vol = volume; EF = ejection fraction; GLS: global longitudinal strain: RV : right ventricular; FWLS: free wall longitudinal strain; LARS: LA reservoir strain; p value refers to male compared to female.

Table 4: Demographics and echocardiographic variables for the five Brazilian regions

		Southeast (1) n= 234 Mean [SD] (95% CI)	Centerwest (2) n= 124 Mean [SD] (95% CI)	Northeast (3) n= 48 Mean [SD] (95% CI)	South (4) n= 45 Mean [SD] (95% CI)	North (5) n= 45 Mean [SD] (95% CI)	p-value
Age (years)		43.5 [16.0] (41.4 - 45.5)	42.0 [14.2] (39.5 - 44.5)	34.9 [11.1] (31.7 - 38.2)	39.5 [14.4] (35.1 - 43.8)	37.4 [13.3] (33.4 - 41.4)	0.003
	Post-hoc	1 - 3	2 - 3	2 - 3; 1 - 3			
Weight (kg)		72.2 [11.8] (70.7 - 73.8)	69.9 [11.6] (67.9 – 72.0)	68.9 [10.5] (65.9 – 72.0)	68.6 [11.4] (65.2 - 72.1)	72.1 [9.8] (69.2 - 75.1)	0.037
	Post-hoc	1 - 5; 1 - 4		3 - 5	1 - 4	1 - 5; 3 - 5	
Height (cm)		168.8 [9.5] (167.6 - 170.1)	166.9 [8.9] (165.3 - 168.5)	167.4 [8.4] (165.0 - 169.9)	164.8 [8.5] (162.3 - 167.4)	167.2 [8.8] (164.6 – 170.0)	0.062
BSA (m^2)		1.82 [0.19] (1.80 - 1.85)	1.78 [0.19] (1.75 - 1.82)	1.78 [0.17] (1.73 - 1.82)	1.75 [0.18] (1.70 - 1.81)	1.81 [0.16] (1.76 - 1.86)	0.027
	Post-hoc	1 - 4			1 - 4		
BMI (kg/m^2)		25.2 [2.9] (24.9 - 25.6)	24.9 [2.6] (24.5 - 25.4)	24.5 [2.7] (23.7 - 25.3)	25.1 [2.5] (24.4 - 25.9)	25.7 [2.9] (24.9 - 26.7)	0.162
Heart Rate (bpm)		68.6 [10.3] (67.3 - 70.0)	71.0 [10.4] (69.2 - 73.0)	70.5 [10.3] (67.5 - 73.5)	69.7 [10.5] (66.6 - 72.9)	67.2 [10.0] (64.2 - 70.3)	0.161
LA (mm)		33.7 [3.3] (33.3 - 34.2)	33.6 [3.3] (33.1 - 34.3)	32.6 [3.3] (31.7 - 33.6)	33.3 [3.6] (32.3 - 34.4)	33.6 [3.1] (32.7 - 34.6)	0.368
LVEDD (mm)		45.5 [4.0] (45.1 - 46.1)	45.1 [3.7] (44.4 - 45.8)	46.0 [3.7] (44.9 - 47.1)	45.3 [3.7] (44.2 - 46.4)	47.2 [3.7] (46.1 - 48.4)	0.028
	Post-hoc		2 - 5			2 - 5	
LVESD (mm)		29.9 [3.0] (29.5 - 30.3)	29.4 [3] (28.9 – 30.0)	30.5 [3.0] (29.6 - 31.4)	29.5 [2.6] (28.7 - 30.4)	30.9 [3.1] (30.0 - 31.9)	0.025
	Post-hoc		2 - 5			2 - 5	
LVEDD index (mm/m^2)		25.2 [2.4] (24.9 - 25.5)	25.5 [2.3] (25.1 - 25.9)	26.0 [2.1] (25.4 - 26.6)	26.0 [2.6] (25.3 - 26.9)	26.2 [2.3] (25.5 - 26.9)	0.002
	Post-hoc	1 - 3;1 - 4;1 - 5		1 - 3	1 - 4	1 - 5	
LVESD index (mm/m^2)		16.5 [1.7] (16.3 - 16.7)	16.5 [1.8] (16.3 - 16.9)	17.2 [1.8] (16.7 - 17.8)	16.9 [1.6] (16.4 - 17.4)	17.2 [1.6] (16.8 - 17.8)	0.004
	Post-hoc	1 - 3;1 - 5	'2 - 5	1 - 3		1 - 5; 2 - 5	
SWt (mm)		8. 8 [1.2] (8.6 - 8.9)	8.8 [1.1] (8.7 - 9.1)	8.5 [1.1] (8.1 - 8.8)	8.7 [1.2] (8.3 – 9.0)	8.5 [1.4] (8.1 - 8.9)	0.275
PWt (mm)		8.2 [1.1] (8.1 - 8.4)	8.1 [1.0] (8.0 - 8.3)	7.7 [0.9] (7.4 – 8.0)	7.8 [1.0] (7.5 - 8.2)	7.9 [1.2] (7.6 - 8.4)	0.006
	Post-hoc	1 - 3		1 - 3			
LV Mass (g)		128.8 [31.9] (124.7 - 132.9)	129.3 [32.7] (123.5 - 135.1)	125.3 [30.7] (116.5 - 134.2)	121.6 [28.4] (113.0 - 130.1)	138.6 [36.1] (127.8 - 149.5)	0.17
LV Mass index (g/m^2)		70.4 [14.3] (68.6 - 72.3)	72.3 [15.4] (69.6 - 75.1)	70.2 [14.2] (66.1 - 74.3)	69.0 [13.0] (65.2 – 73.0)	76.2 [16.4] (71.2 - 81.1)	0.195
LA Vol (ml)		43.9 [9.7] (42.6 - 45.1)	41.7 [9.9] (39.9 - 43.5)	41.6 [10.8] (38.4 - 44.7)	45.7 [10.4] (42.6 - 48.9)	44.1 [10.1] (41.1 - 47.2)	0.136
LA Vol index (ml/m^2)		24.1 [4.7] (23.5 - 24.7)	23.4 [5.1] (22.5 - 24.4)	23.3 [5.2] (21.8 - 24.8)	26.1 [5.4] (24.4 - 27.7)	24.5 [5.4] (22.8 - 26.1)	0.102
LVED Vol (ml)		88.6 [21.1] (85.9 - 91.4)	81.0 [19.6] (77.6 - 84.6)	91.0 [21.4] (84.7 - 97.4)	92.5 [15.0] (87.9 - 97.1)	94.5 [22.1] (87.8 - 101.2)	<0.001
	Post-hoc	1 - 2	1 - 2; 2 - 3; 2 - 4; 2 - 5	2 - 3	2 - 4	2 - 5	
LVES Vol (ml)		33.4 [9.6] (32.2 - 34.6)	31.1 [10.0] (29.3 - 32.9)	34.7 [8.4] (32.2 - 37.2)	33.9 [7.7] (31.6 - 36.3)	35.6 [9.8] (32.7 - 38.7)	0.017
	Post-hoc	1 - 2	1 - 2; 2 - 3; 2 - 4; 2 - 5	2 - 3	2 - 4	2 - 5	

LVED Vol index (ml/m^2)		48.4 [9.0] (47.2 - 49.6)	45.2 [8.7] (43.7 - 46.8)	51.0 [10.6] (47.9 - 54.2)	53.0 [9.1] (50.2 - 55.8)	52.1 [10.7] (48.8 - 55.3)	<0.001
	Post-hoc	1 - 2; 1 - 4	1 - 2; 2 - 3; 2 - 4; 2 - 5	2 - 3	2 - 4; 1 - 4	2 - 5	
LVES Vol index (ml/m^2)		18.2 [4.2] (17.6 - 18.7)	17.3 [4.6] (16.5 - 18.1)	19.5 [4.3] (18.2 - 20.8)	19.4 [4.4] (18.1 - 20.8)	19.6 [4.8] (18.2 - 21.1)	0.003
	Post-hoc		2 - 3; 2 - 4; 2 - 5	2 - 3	2 - 4	2 - 5	
Stroke Vol (ml)		55. [13.4] (53.4 - 56.8)	49.9 [11.4] (47.9 - 51.9)	55.3 [14.8] (50.9 - 59.7)	58.2 [8.8] (55.6 - 60.9)	55.9 [12.9] (52.1 - 59.9)	<0.001
	Post-hoc	1 - 2	1 - 2; 2 - 4; 2 - 5				
Stroke Vol index (ml/m^2		30.2 [6.1] (29.4 - 30.9)	27.9 [5.5] (27.0 - 28.9)	30.9 [7.2] (28.8 - 33.1)	33.4 [5.5] (31.7 - 35.1)	30.9 [6.5] (28.9 - 32.8)	<0.001
	Post-hoc	1 - 2; 1 - 4	1 - 2; 2 - 3; 2 - 4; 2 - 5	2 - 3; 3 - 4	1 - 4; 2 - 4; 3 - 4	2 - 5	
LVEF (%)		62.5 [4.6] (61.9 - 63.1)	62.2 [4.9] (61.3 - 63.1)	61.7 [3.8] (60.6 - 62.8)	63.6 [4.3] (62.3 - 64.9)	61.3 [3.9] (60.1 - 62.5)	0.186
LV GLS		21.4 [2.9] (21.0 - 21.8)	19.9 [1.6] (19.6 - 20.3)	21.3 [1.5] (20.9 - 21.9)	22.9 [2.3] (22.1 - 23.7)	20.7 [2.0] (20.1 - 21.4)	<0.001
	Post-hoc	1 - 2; 1 - 4	1 - 2; 2 - 3; 2 - 4; 2 - 5	2 - 3; 3 - 4	1 - 4; 2- 4; 4- 5; 3 - 4	2 - 5; 4- 5	
RV GLS		22.9 [2.5] (22.6 - 23.3)	21.6 [2.2] (21.2 – 22.0)	23.9 [3.1] (23.0 – 25.0)	23.7 [2.7] (22.8 - 24.6)	22.2 [2.2] (21.6 – 23.0)	<0.001
	Post-hoc	1 - 2	1 - 2; 2 - 3; 2 - 4	2 - 3; 3 - 5	2 - 4; 4 - 5	3 - 5; 4 - 5	
RVFWLS		27.3 [3.5] (26.9 - 27.9)	26.0 [3.4] (25.4 - 26.7)	27.6 [3.9] (26.4 - 28.8)	27.9 [3.0] (27.0 – 29.0)	26.0 [3.8] (24.9 - 27.3)	<0.001
	Post-hoc	1 - 2	1 - 2; 2 - 3; 2 - 4	2 - 3; 3 - 5	2 - 4; 4 - 5	4 - 5	
LARS		40.8 [6.4] (40.0 - 41.7)	40.3 [5.3] (39.4 - 41.3)	43.1 [5.1] (41.5 - 44.7)	40.8[4.9] (39.3 - 42.4)	39.5 [4.1] (38.2 - 40.8)	0.043
	Post-hoc	1 - 3	2 - 3	1 - 3; 2 - 3; 3 - 5		3 - 5	

BSA: body surface area; BMI = body mass index; LA = left atrium; LV = left ventricular; ED = end-diastolic; D = diameter; ES = end- systolic; SWt = septal wall thickness; PWt = posterior wall thickness; Vol = volume; index = indexed; EF =ejection fraction; GLS: global longitudinal strain: RV = right ventricular; FWLS= free wall longitudinal strain; LARS= LA reservoir strain; SD = standard deviation; CI= confidence interval; p value refers to male compared to female; *Post-hoc:* Benjamini-Hochberg or Duncan.

Table 5: Demographics and echocardiographic variables for the five Brazilian regions for male (n= 224) and female (n = 272).

		Southeast (1) n= 109 Mean [SD] (95% CI)	Centerwest (2) n= 57 Mean [SD] (95% CI)	Northeast (3) n= 21 Mean [SD] (95% CI)	South (4) n= 13 Mean [SD] (95% CI)	North (5) n= 24 Mean [SD] (95% CI)	p-value
Age (years)	Male	42.3 [15.6] (39.3 - 45.3)	40.9 [13.5] (37.4 - 44.6)	33.7 [10.4] (29.0 - 38.5)	44.0 [17.4] (33.4 - 54.6)	35.3 [12.6] (30.0 - 40.7)	0.061
	Female	44.4 [16.4] (41.5 - 47.3)	42.9 [14.8] (39.3 - 46.5)	35.8 [11.7] (31.3 - 40.5)	37.6 [12.8] (33 - 42.3)	39.7 [13.9] (33.4 – 46.0)	0.041
	Female *Post hoc*			3 - 4	3 - 4		
Weight (kg)	Male	78.4 [9.9] (76.6 - 80.3)	77.8 [9.4] (75.4 - 80.3)	75.2 [10.4] (70.5 - 79.9)	78.9 [15.1] (69.8 – 88.0)	77.1 [8.8] (73.4 - 80.8)	0.714
	Female	66.8 [10.7] (64.9 - 68.7)	63.2 [8.7] (61.1 - 65.3)	64.1 [7.8] (61.0 - 67.1)	64.4 [5.9] (62.3 - 66.6)	66.4 [7.7] (62.9 - 69.9)	0.116
Height (cm)	Male	175.2 [7.8] (173.7 - 176.7)	173.5 [5.5] (172.0 – 175.0)	172.8 [7.3] (169.5 - 176.2)	173 [10.4] (166.7 - 179.3)	173.7 [5.8] (171.3 - 176.2)	0.42

	Female	163.3 [6.9]<br>(162.0 - 164.5)	161.3 [7.3]<br>(159.6 - 163.1)	163.2 [6.7]<br>(160.6 - 165.9)	161.5 [4.8]<br>(159.8 - 163.3)	159.9 [5.3]<br>(157.5 - 162.4)	0.117
BSA<br>(m^2)	Male	1.94 [0.15]<br>(1.91 - 1.97)	1.92 [0.13]<br>(1.89 - 1.96)	1.89 [0.16]<br>(1.81 - 1.96)	1.93 [0.24]<br>(1.78 - 2.07)	1.91 [0.11]<br>(1.87 - 1.96)	0.754
	Female	1.71<br>(1.69 - 1.75)	1.66<br>(1.63 - 1.70)	1.69<br>(1.65 - 1.73)	1.69<br>(1.65 - 1.71)	1.70<br>(1.64 - 1.74)	0.186
BMI<br>(kg/m^2)	Male	25.5 [2.6]<br>(25.0 – 26.0)	25.8 [2.3]<br>(25.2 - 26.4)	25.0 [2.4]<br>(24.0 - 26.2)	26.1 [2.4]<br>(24.7 - 27.6)	25.6 [2.9]<br>(24.4 - 26.8)	0.702
	Female	24.9 [3.1]<br>(24.4 - 25.5)	24.2 [2.6]<br>(23.6 - 24.9)	24.1 [2.9]<br>(22.9 - 25.2)	24.7 [2.5]<br>(23.8 - 25.6)	25.9 [3.1]<br>(24.6 - 27.4)	0.108
Heart Rate<br>(bpm)	Male	66.5 [9.6]<br>(64.6 - 68.3)	69.1 [10.5]<br>(66.3 - 71.9)	68.1 [10.7]<br>(63.2 - 72.9)	69.8 [12.9]<br>(62.0 - 77.7)	65.3 [9.8]<br>(61.2 - 69.5)	0.161
	Female	70.5 [10.7]<br>(68.6 - 72.4)	72.8 [10.2]<br>(70.3 - 75.3)	72.3 [9.9]<br>(68.4 - 76.2)	69.7 [9.6]<br>(66.2 - 73.1)	69.3 [9.8]<br>(64.9 - 74)	0.372
LA (mm)	Male	35.1 [3.3]<br>(34.5 - 35.7)	35.4 [2.9]<br>(34.7 - 36.2)	34.3 [2.7]<br>(33.0 - 35.5)	34.5 [4.2]<br>(31.9 – 37.0)	34.4 [3.2]<br>(33.1 - 35.8))	0.368
	Female	32.5 [2.9]<br>(32.0 - 33.1)	32.2 [2.9]<br>(31.5 - 32.9)	31.4 [3.2]<br>(30.2 - 32.7)	32.9 [3.3]<br>(31.7 - 34.1)	32.9 [2.9]<br>(31.5 - 34.2)	0.293
LVEDD<br>(mm)	Male	47.5 [3.6]<br>(46.8 - 48.2)	47.2 [3.6]<br>(46.3 - 48.2)	47.9 [3.4]<br>(46.4 - 49.5)	46.6 [4.1]<br>(44.1 - 49.1)	48.3 [3.3]<br>(46.9 - 49.7)	0.613
	Female	43.9 [3.5]<br>(43.3 - 44.5)	43.31 [2.7]<br>(42.7 – 44.0)	44.5 [3.3]<br>(43.2 - 45.8)	44.7 [3.43]<br>(43.5 – 46.0)	46.1 [3.9]<br>(44.3 - 47.8)	0.013
	Female<br>*Post-hoc*	1 - 5	2 - 5		4 - 5	1 - 5; 2 - 5;<br>4 - 5	
LVESD<br>(mm)	Male	31.2 [2.9]<br>(30.7 - 31.8)	30.8 [3.1]<br>(30.0 - 31.6)	31.5 [3.2]<br>(30.1 – 33.0)	30.6 [3.1]<br>(28.8 - 32.5)	31.8 [3.3]<br>(30.4 - 33.2)	0.62
	Female	28.8 [2.7]<br>(28.3 - 29.3)	28.2 [2.3]<br>(27.7 - 28.8)	29.7 [2.7]<br>(28.6 - 30.8)	29.1 [2.4]<br>(28.2 – 30.0)	30 [2.6]<br>(28.8 - 31.2)	0.040
	Female<br>*Post-hoc*	1 - 5	2 - 5. 2 - 3	2 - 3		1 - 5; 2 - 5	
LVEDD<br>index<br>(mm/m^2)	Male	24.6 [2.1]<br>(24.2 – 25.0)	24.7 [2.1]<br>(24.1 - 25.3)	25.5 [1.8]<br>(24.7 - 26.4)	24.5 [2.8]<br>(22.9 - 26.2)	25.2 [1.9]<br>(24.4 - 26.1)	0.356
	Female	25.6 [2.6]<br>(25.2 - 26.1)	26.1 [2.2]<br>(25.6 - 26.6)	26.4 [2.2]<br>(25.5 - 27.3)	26.7 [2.2]<br>(25.9 - 27.6)	27.3 [2.1]<br>(26.4 - 28.3)	0.009
	Female<br>*Post-hoc*	1 - 5; 1 - 4	2 - 5		1 - 4	1 - 5; 2 - 5	
LVESD<br>index<br>(mm/m^2)	Male	16.2 [1.5]<br>(15.9 - 16.5)	16.1 [1.9]<br>(15.6 - 16.6)	16.7 [1.7]<br>(16.0 - 17.5)	15.8 [1.3]<br>(15.1 - 16.6)	16.8 [1.7]<br>(16.1 - 17.5)	0.226
	Female	16.8 [1.9]<br>(16.5 - 17.1)	16.9 [1.6]<br>(16.6 - 17.4)	17.6 [1.9]<br>(16.9 - 18.4)	17.3 [1.5]<br>(16.8 - 17.9)	17.8 [1.3]<br>(17.2 - 18.5)	0.014
	Female<br>*Post-hoc*	1 - 5; 1 - 3	2 - 5	1 - 3		1 - 5; 2 - 5	
SWt<br>(mm)	Male	9.2 [1.1]<br>(9.0 - 9.4)	9.3 [1.2]<br>(9 - 9.6)	9.0 [0.9]<br>(8.6 - 9.4)	9.5 [1.3]<br>(8.7 - 10.2)	9.3 [1.4]<br>(8.7 - 9.8)	0.808
	Female	8.4 [1.1]<br>(8.2 - 8.6)	8.4 [0.9]<br>(8.3 - 8.7)	8.0 [1.1]<br>(7.6 - 8.5)	8.3 [1]<br>(8.0 - 8.7)	7.6 [1.0]<br>(7.2 - 8.1)	0.006
	Female<br>*Post-hoc*	1 - 5	2 - 5; 2 - 3	2 - 3	4 - 5	1 - 5; 2 - 5;<br>4 - 5	
PWt<br>(mm)	Male	8.6 [1.0]<br>(8.4 - 8.8)	8.6 [1.0]<br>(8.3 - 8.9)	8.0 [0.9]<br>(7.6 - 8.5)	8.8 [1.0]<br>(8.2 - 9.4)	8.5 [1.3]<br>(8.0 - 9.1)	0.346
	Female	7.9 [1.0]<br>(7.7 - 8.1)	7.8 [0.9]<br>(7.6 – 8.0)	7.4 [0.9]<br>(7.1 - 7.8)	7.5 [0.8]<br>(7.2 - 7.8)	7.3 [0.7]<br>(7.0 - 7.7)	0.005
	Female<br>*Post-hoc*	1 - 3; 1 - 4;<br>1 - 5	2 - 5	1 - 3	1 - 4	1 - 5; 2 - 5	
LV Mass<br>(g)	Male	145.5 [32.0]<br>(139.4 - 151.6)	148.4 [33.5]<br>(139.5 - 157.3)	141.6 [27.3]<br>(129.1 – 154.0)	147.4 [31.3]<br>(128.4 - 166.3)	156.4 [35.9]<br>(141.2 - 171.6)	0.522
	Female	114.2 [23.5]<br>(110.0 - 118.3))	113.0 [21.4]<br>(107.8 - 118.3)	112.7 [27.1]<br>(102.0 - 123.5)	111.1 [19.3]<br>(104.1 – 118.0)	118.4 [24.1]<br>(107.4 - 129.3)	0.78
LV Mass<br>index	Male	75.0 [15.7]<br>(72.1 – 78.0)	77.3 [16.9]<br>(72.9 - 81.9)	74.8 [11.1]<br>(69.7 - 79.9)	76.7 [14.9]<br>(67.6 - 85.6)	81.3 [15.9]<br>(74.6 – 88.0)	0.418

(g/m2)							
	Female	66.4 [11.9] (64.3 - 68.5)	68 [12.7] (64.9 - 71.1)	66.6 [15.2] (60.6 - 72.7)	66.0 [10.9] (62.1 – 70.0)	70.3 [15.2] (63.4 - 77.2)	0.806
LA Vol (ml)	Male	46.8 [10.4] (44.8 - 48.8)	45.0 [10.2] (42.3 - 47.8)	47.2 [11.2] (42.1 - 52.3)	48.9 [10.1] (42.8 – 55.0)	45.8 [10.6] (41.3 - 50.3)	0.724
	Female	41.3 [8.3] (39.9 - 42.8)	38.9 [8.8] (36.8 - 41.1)	37.0 [8.3] (33.7 - 40.4)	44.4 [10.4] (40.6 - 48.2)	42.2 [9.4] (38.0 - 46.5)	0.022
	Female *Post-hoc*	1 - 3	2 - 4	1 - 3; 3 - 4; 3 - 5	2-4; 3 - 4	2- 5	
LA Vol index (ml/m^2)	Male	24.1 [4.9] (23.2 - 25.1)	23.5 [5.2] (22.1 - 24.9)	24.9 [5.2] (22.6 - 27.3)	25.5 [4.6] (22.7 - 28.3)	23.9 [5.4] (21.7 - 26.3)	0.684
	Female	24.1 [4.4] (23.3 - 24.8)	23.4 [5.1] (22.1 - 24.6)	21.9 [4.9] (20.0 - 23.9)	26.3 [5.8] (24.2 - 28.5)	25.0 [5.4] (22.6 - 27.5)	0.035
	Female *Post-hoc*	1 - 3	2-4	1- 3; 3 - 4; 3 - 5	2-4;3 - 4	2 - 5	
LVED Vol (ml)	Male	101.6 [20.3] (97.7 - 105.4)	93.4 [17.7] (88.7 - 98.1)	105.0 [20.5] (95.7 - 114.4)	96.7 [13.9] (88.2 - 105.1)	106.4 [20.8] (97.6 - 115.2)	0.062
	Female	77.4 [14.3] (74.8 - 79.9)	70.6 [14.4] (67.1 - 74.1)	79.2 [13. 9] (73.5 – 85.0)	90.7 [15.3] (85.1 - 96.4)	80.2 [13.7] (73.8 - 86.6)	<0.001
	Female *Post-hoc*	1 - 2; 1 - 4	1 - 2; 2 - 3; 2 - 4; 2 - 5	3 - 4	1 - 4; 2 - 4; 3 - 4; 4 - 5	4 - 5	
LVES Vol (ml)	Male	38.9 [9.5] (37.1 - 40.7)	37.7 [9.7] (35.1 - 40.3)	39.7 [7.8] (36.2 - 43.3)	36.2 [7.9] (31.5 – 41.0)	40.2 [10.3] (35.9 - 44.6)	0.539
	Female	28.6 [6.6] (27.4 - 29.8)	25.5 [6.2] (24.0 – 27.0)	30.4 [6.3] (27.8 – 33.0)	33.0 [7.6] (30.2 - 35.8)	30.2 [5.9] (27.4 – 33.0)	<0.001
	Female *Post -hoc*	1 - 2; 1 - 4	1 - 2; 2 - 3; 2 - 4; 2 - 5	2 - 3	2 - 4	2 - 5	
LVED Vol index (ml/m^2)	Male	52.2 [8.9] (50.6 - 53.9)	48.7 [8.9] (46.3 – 51.0)	55.8 [10.9] (50.9 - 60.8)	50.6 [7.4] (46.2 - 55.1)	55.8 [11.3] (51.0 - 60.6)	0.029
	Male *Post-hoc*		2 - 3; 2 - 5	2 - 3		2 - 5	
	Female	45.1 [7.8] (43.7 - 46.4)	42.3 [7.4] (40.5 - 44.1)	47 [8.6] (43.5 - 50.5)	54.0 [9.7] (50.5 - 57.6)	47.6 [7.9] (43.9 - 51.3)	<0.001
	Female *Post-hoc*	1 - 2; 1 - 4	1 - 2; 2 - 3; 2 - 4; 2 - 5	3 - 4	1 - 4; 2 - 4; 3 - 4; 4 - 5	4 - 5	
LVES Vol index (ml/m^2)	Male	20.0 [4.1] (19.2 - 20.8)	19.6 [4.8] (18.3 - 20.9)	21.2 [4.4] (19.2 - 23.2)	18.8 [3.4] (16.8 - 20.9)	21.0 [5.3] (18.8 - 23.3)	0.334
	Female	16.6 [3.7] (16.0 - 17.3)	15.3 [3.3] (14.5 - 16.1)	18.2 [3.8] (16.5 - 19.6)	19.7 [4.9] (17.9 - 21.5)	17.9 [3.4] (16.3 - 19.5)	<0.001
	Female *Post-hoc*	1 - 2; 1 - 4	1 - 2; 2 - 3; 2 - 4; 2 - 5	2 - 3	2 - 4	2 - 5	
Stroke Vol (ml)	Male	62.4 [13.1] (59.9 - 64.9)	55.5 [10.2] (52.8 - 58.3)	64.4 [15.3] (57.4 - 71.4)	58.7 [9.4] (53.0 - 64.4)	62.2 [11.9] (57.2 - 67.3)	0.025
	Male *Post-hoc*	1 - 2	1 - 2; 2 - 3; 2 - 5	2 - 3		2 - 5	
	Female	48.7 [9.9] (47.0 - 50.5)	45.1 [10.2] (42.6 - 47.6)	47.6 [9.1] (43.8 - 51.4)	58.0 [8.7] (54.8 - 61.2)	48.5 [9.9] (43.8 - 53.1)	<0.001
	Female *Post-hoc*	1 - 2; 1 - 4	1 - 2; 2 - 4	3 - 5	4 - 5	3 - 5; 4 - 5	
Stroke Vol index (ml/m^2)	Male	32.1 [6.2] (31.0 - 33.3)	29.0 [5.4] (27.6 - 30.4)	34.11 [7.7] (30.6 - 37.6)	30.9 [5.7] (27.4 - 34.4)	32.6 [6.5] (29.9 - 35.4)	0.015
	Male *Post-hoc*	1 - 2	1 - 2; 2 - 3; 2 - 5	2 - 3		2 - 5	
	Female	28.4 [5. 6] (27.4 - 29.4)	27.0 [5.5] (25.7 - 28.4)	28.2 [5.6] (25.9 - 30.6)	34.5 [5.2] (32.6 - 36.4)	28.8 [5.2] (26.0 - 31.6)	<0.001
	Female *Post-hoc*	1 - 2; 1 - 4	1 - 2; 2 - 4	3 - 5	4 - 5	3 - 5; 4 - 5	
LVEF (%)	Male	61.7 [4.3] (60.9 - 62.6)	60.4 [4.5] (59.2 - 61.6)	61.7 [3.5] (60.2 - 63.4)	63.0 [4.2] (60.6 - 65.6)	61.2 [4.4] (59.3 – 63.0)	0.208
	Female	63.1 [4.8] (62.3 – 64.0)	63.7 [4.7] (62.6 - 64.9)	61.6 [4.2] (59.9 - 63.4)	63.8 [4.4] (62.2 - 65.4)	61.5 [3.4] (60.0 - 63.1)	0.233

LV GLS	Male	20.9 [1.9] (20.5 - 21.3)	19.3 [1.2] (19.0 - 19.6)	21 [1.2] (20.4 - 21.6)	21.8 [2.3] (20 - 23.6)	20.7 [1.9] (19.8 - 21.6)	<0.001
	Male *Post-hoc*		2 - 5			2 - 5	
	Female	21.9 [3.6] (21.2 - 22.6)	20.4 [1.8] (19.9 - 20.9)	21.7 [1.7] (20.9 - 22.5)	23.3 [2.3] (22.4 - 24.2)	20.8 [2.1] (19.7 - 21.8)	<0.001
	Female *Post-hoc*	1 - 2;1- 4	1 - 2; 2 - 3; 2 - 4	2 - 3	2 - 4;1- 4;	4 - 5	
RV GLS	Male	22.4 [2.3] (22.0 - 22.9)	21.4 [2.2] (20.7 – 22.0)	23.5 [2.8] (22.1 - 24.9)	22.6 [4.1] (19.7 - 25.6)	22.2 [2.0] (21.3 – 23.0)	0.033
	Male *Post-hoc*	1 - 2	1 - 2; 2 - 3; 2 - 4; 2 - 5	2 - 3	2 - 4	2 - 5	
	Female	23.6 [2.8] (22.9 - 24)	21.8 [2.2] (21.2 - 22.4)	24.3 [3.4] (23 - 25.8)	24.1 [1.9] (23.3 - 24.8)	22.4 [2.5] (21.2 - 23.6)	<0.001
	Female *Post-hoc*	1 - 2;	1 - 2; 2 - 3; 2 - 4	2 - 3	2 - 4; 4 - 5	4 - 5	
RVFWLS	Male	26.8 [3.3] (24.9 - 28.7)	25.8 [3.3] (24.9 - 26.7)	26.8 [3.8] (23.6 - 29.2)	26.4 [3.6] (26.1 - 27.5)	26.0 [4.0] (24.2 - 27.8)	0.471
	Female	27.9 [3.7] (27.1 - 28.6)	26.2 [3.6] (25.3 - 27.1)	28.2 [3.9] (26.6 - 29.8)	28.4 [2.7] (27.4 - 29.5)	26.2 [3.6] (24.3 – 28.0)	0.008
	Female *Post-hoc*	1 - 2; 1 - 5	1 - 2; 2 - 3; 2 - 4	2 - 3	4 - 5	1 - 5; 4 - 5	
LARS	Male	39.9 [6.6] (38.6 - 41.2)	40.2 [5.5] (38.7 - 41.7)	42.5 [4.7] (40.3 - 44.7)	40.9 [5.1] (37.7 - 44.1)	40.3 [3.3] (38.8 - 41.8)	0.534
	Female	41.7 [6.3] (40.6 - 42.8)	40.5 [5.3] (39.2 - 41.8)	43.7 [5.5] (41.2 - 46.1)	40.8 [4.9] (38.9 - 42.8)	38.6 [4.8] (36.2 – 41.0)	0.049
	Female *Post-hoc*	1 - 5	2 - 3	3 - 5		1 - 5; 3 - 5	

BSA: body surface area; BMI = body mass index; LA = left atrium; LV = left ventricular ED = end-diastolic; D = diameter; ES = end- systolic; SWt = septal wall thickness; PWt = posterior wall thickness; Vol = volume; index = indexed; EF =ejection fraction; SD= standard deviation; CI= confidence interval; p value refers to male compared to female; *Post-hoc*: Benjamini-Hochberg or Duncan test as applicable.

Table 6: Demographics and echocardiographic variables of Mixed-race and White individuals

	All (n= 434) Mean [SD] (95% CI)	Mixed-race (n= 157) Mean [SD] (95% CI)	White (n=277) Mean [SD] (95% CI)	p-value
Age (years)	40.8 [14.8] (39.5 - 42.3)	37.7 [13.7] (35.5 - 39.8)	42.7 [15.1] (40.9 - 44.5)	<0.001
Weight (kg)	70.8 [11.5] (69.7 - 71.9)	70.3 [11.1] (68.5 – 72.0)	71.1 [11.7] (69.8 - 72.5)	0.448
Height (cm)	167.7 [9.2] (166.9 - 168.6)	166.8 [8.8] (165.5 - 168.2)	168.3 [9.6] (167.2 - 169.4)	0.12
BSA (m^2)	1.80 [0.10] (1.78 - 1.81)	1.79 [0.17] (1.76 - 1.81)	1.81 [0.19] (1.78 - 1.83)	0.255
BMI (kg/m^2)	25.1 [2.8] (24.8 - 25.3)	25.2 [2.9] (24.7 - 25.6)	25.0 [2.7] (24.7 - 25.3)	0.634
Heart Rate (bpm)	69.4 [10.4] (68.4 - 70.4)	69.5 [10.8] (67.8 - 71.2)	69.3 [10.1] (68.1 - 70.5)	0.918
LA (mm)	33. 6 [3.4] (33.2 - 33.9)	33.4 [3.3] (32.9 - 33.9)	33.7 [3.3] (33.3 - 34.1)	0.483
LVEDD (mm)	45.7 [3.9] (45.3 – 46.0)	45.9 [3.8] (45.3 - 46.5)	45.5 [3.9] (45.1 – 46.0)	0.373
LVESD (mm)	29.9 [3.0] (29.7 - 30.2)	30.0 [.9] (29.5 - 30.5)	29.9 [3.1] (29.5 - 30.3)	0.835

LVEDD index(mm/m^2)	25.6 [2.4] (25.3 - 25.8)	25.8 [2.3] (25.5 - 26.2)	25.4 [2.4] (25.1 - 25.7)	0.102
LVESD index (mm/m^2)	16.7 [1.8] (16.6 - 16.9)	16. 9 [1. 7] (16.6 - 17.1)	16.7 [1.8] (16.4 - 16.9)	0.186
SWt (mm)	8.7 [1.2] (8.6 - 8.8)	8.6 [1.1] (8.4 - 8.8)	8.73 [1.2] (8.6 - 8.9)	0.26
PWt (mm)	8.1 [1.1] (8.0 - 8.2)	8.1 [1.1] (7.9 - 8.2)	8.1 [1.1] (8.0- 8.2)	0.908
LV Mass (g)	128.6 [32.5] (125.6 - 131.7)	129.7 [31.5] (124.8 - 134.7)	128.0 [33.2] (124.1 - 131.9)	0.407
LV Mass index (g/m^2)	71.2 [14.9] (69.8 - 72.6)	72.4 [14.3] (70.1 - 74.6)	70.5 [15.2] (68.7 - 72.3)	0.137
LA Vol (ml)	43.5 [10.0] (42.5 - 44.4)	43.4 [10.1] (41.8 – 45.0)	43.5 [10.1] (42.3 - 44.7)	0.982
LA Vol index (ml/m)	24.2 [5.0] (23.7 - 24.6)	24.3 [5.2] (23.5 - 25.1)	24.1 [4.9] (23.5 - 24.7)	0.62
LVED Vol (ml)	88.1 [21.0] (86.1 - 90.1)	89.9 [21.3] (86.6 - 93.3)	87.1 [20. 9] (84.6 - 89.6)	0.159
LVES Vol (ml)	33.4 [9.64] (32.4 - 34.3)	33.8 [9.7] (32.3 - 35.3)	33.1 [9.6] (32.0 - 34.2)	0.411
LVED Vol index (ml/m^2)	48.8 [9.8] (47.9 - 49.7)	50.2 [10.1] (48.6 - 51.8)	48.0 [9.5] (46.9 - 49.2)	0.025
LVES Vol index (ml/m^2)	18.4 [4.5] (18.0 - 18.9)	18.8 [4.5] (18.1 - 19.5)	18.2 [4.5] (17.7 - 18.7)	0.131
Stroke Vol (ml)	54.2 [13.0] (53.0 - 55.5)	55.1 [12.8] (53.0 - 57.1)	53.8 [13.2] (52.2 - 55.3)	0.243
Stroke Vol index (ml/m^2)	30.1 [6.3] (29.5 - 30.7)	30.8 [6.4] (29.8 - 31.8)	29.7 [6.3] (29.0 - 30.4)	0.084
LVEF (%)	62.2 [4.6] (61.8 - 62.7)	62.1 [4.6] (61.4 - 62.9)	62.3 [4.6] (61.8 - 62.9)	0.728
LV GLS (%)	21.1 [2.5] (20.8 - 21.4)	21.2 [2.0] (20.9 - 21.6)	21.0 [2.9] (20.7 - 21.4)	0.095
RV GLS (%)	22.7 [2.6] (22.5 – 23.0)	23.2 [2.6] (22.8 - 23.7)	22.4 [2.5] (22.1 - 22.8)	0.005
RVFWLS (%)	26.9 [3.6] (26.7 - 27.4)	27.5 [3.7] (26.9 - 28.2)	26.7 [3.4] (26.4 - 27.2)	0.099
LARS (%)	40.8 [5.8] (40.3 - 41.4)	41.7 [5.3] (40.9 - 42.7)	40.3 [5.8] (39.6 – 41.0)	0.016

LA = left atrium; BSA= body surface area; BMI = body mass index; LV = left ventricular ED = end-diastolic; D = diameter; ES = end- systolic; SWt = septal wall thickness; PWt = posterior wall thickness; Vol = volume; index= indexed; EF = ejection fraction; GLS= global longitudinal strain; RV= right ventricular; FWLS= free wall longitudinal strain; LARS= LA reservoir strain: SD= standard deviation; CI= confidence interval; p value refers to white compared to mixed-race. Student's t test or Mann-Whitney test when applicable
