# Supplementary material for: Valores Ecocardiográficos de Referência para as Câmaras Cardíacas no Brasil: Um Estudo Multirregional e Multirracial
Source: Arq Bras Cardiol. 2026 Mar 3;123(2):e20250628. [Article in Portuguese] doi: 10.36660/abc.20250628 (PMC13128182; doi:10.36660/abc.20250628)
Supplement: Translation

# Translation: Reference Echocardiographic Values for Cardiac Chambers in Brazil: A Multiregional, Multi-Racial Study

**DOI:** 10.36660/abc.20250628i

**Authors:** Ana Clara Tude Rodrigues, Roberto Magalhães Saraiva, Viviane T. Hotta, Gabriel Doreto Rodrigues, Fabiana Camelier de Assis Cardoso, Anderson da Costa Armstrong, Cláudia Gianini Monaco, Adelino Parro, José Luis de Castro e Silva Pretto, João Marcos Bemfica Barbosa Ferreira, Renata Santos Lopes Teixeira de Lima, Marcelo Salame, Salustiano Pereira de Araujo, Carlos Eduardo Suaide Silva, Cíntia Galhardo Tressino, Fabio Canellas Moreira, Ana Cristina Camarozano, Minna Moreira Dias Romano, Andre Luiz Cerqueira Almeida, Claudio Henrique Fischer, Alex dos Santos Felix, Clarissa Borguezan-Daros, Diogo Silva Piardi, Diego Cunha, Marcelo Dantas Tavares de Melo, Rafael Modesto, Silvio Henrique Barberato, Wercules Antonio Oliveira, Marcelo Luiz Campos Vieira, Samira Saady Morhy

**Affiliations:** 1 Brazil Hospital Israelita Albert Einstein, São Paulo, SP – Brazil; 2 Brazil Instituto de Radiologia do Hospital das Clínicas da Faculdade de Medicina da Universidade de São Paulo (INRAD HCFMUSP), São Paulo, SP – Brazil; 3 Brazil Fundação Oswaldo Cruz, Rio de Janeiro, RJ – Brazil; 4 Brazil Fleury Medicina e Saúde, Grupo Fleury, São Paulo, SP – Brazil; 5 Brazil Hospital Universitário Maria Aparecida Pedrossian, Universidade Federal de Mato Grosso do Sul, Ebserh, Campo Grande, MS – Brazil; 6 Brazil Instituto D’Or de Pesquisa e Ensino (IDOR), Hospital CardioPulmonar Salvador, BA – Brazil; 7 Brazil Universidade Federal do Vale do São Francisco Curso de Medicina, Petrolina, PE – Brazil; 8 Brazil Instituto de Moléstias Cardiovasculares (IMC), São José do Rio Preto, SP – Brazil; 9 Brazil Hospital São Vicente de Paulo, Passo Fundo, RS – Brazil; 10 Brazil Universidade do Estado do Amazonas, Manaus, AM – Brazil; 11 Brazil Hospital Amecor, Cuiabá, MS – Brazil; 12 Brazil Jipamed Medicina Avançada, Ji-Paraná, RO – Brazil; 13 Brazil Hospital Santa Genoveva, Uberlândia, MG – Brazil; 14 Brazil Diagnósticos da América SA, Barueri, SP – Brazil; 15 Brazil Instituto Dante Pazzanese de Cardiologia Ecocardiografia, São Paulo, SP – Brazil; 16 Brazil Santa Casa de Misericórdia de Porto Alegre, Porto Alegre, RS – Brazil; 17 Brazil Hospital de Clínicas da Universidade Federal do Paraná, Curitiba, PR – Brazil; 18 Brazil Universidade de São Paulo Hospital das Clínicas da Faculdade de Medicina de Ribeirão Preto, Ribeirão Preto, SP – Brazil; 19 Brazil Santa Casa de Misericórdia de Feira de Santana Cardiologia, Feira de Santana, BA – Brazil; 20 Brazil Instituto Nacional de Cardiologia, Rio de Janeiro, RJ – Brazil; 21 Brazil Hospital São José, Criciúma, SC – Brazil; 22 Brazil Universidade Federal da Paraíba, João Pessoa, PB – Brazil; 23 Brazil Hospital Aliança, Salvador, BA – Brazil; 24 Brazil CardioEco Centro de Diagnóstico Cardiovascular, Curitiba, PR – Brazil; 25 Brazil Quanta Diagnóstico Ecocardiografia, Curitiba, PR – Brazil; 26 Brazil Instituto do Coração do Hospital das Clínicas da Faculdade de Medicina da Universidade de São Paulo, São Paulo, SP – Brazil

**Keywords:** Echocardiography, Heart, Reference Values

## Abstract

**Background:**

Reference values for cardiac chamber quantification are essential for clinical decision-making. Brazil is a continental country with marked racial diversity and substantial regional socioeconomic and anthropometric variability, which may not be adequately represented in international echocardiographic standards.

**Objective:**

To establish reference echocardiographic measurements for healthy Brazilian adults and to investigate geographic and sex-related differences across Brazil’s major regions.

**Methods:**

In this prospective, multicenter study, transthoracic echocardiograms were performed in 496 healthy volunteers (mean age 41 ± 15 years; 55% women) from five Brazilian regions. Left ventricular (LV) and left atrial (LA) dimensions, volumes, mass, ejection fraction, and global longitudinal strain (GLS) were analyzed in accordance with current guidelines. Comparisons were conducted by sex (Student’s t test or Mann-Whitney U test) and by region (analysis of variance).

**Results:**

Cardiac chamber dimensions and LV mass were greater in men, even after indexing for body surface area. LA volumes were also higher in men, although these differences were attenuated after indexing. Absolute LVGLS and right ventricle (RV) GLS (RVGLS) values were higher in women, whereas LA strain reservoir (LASr) was similar between sexes. Regionally, the Center-West exhibited significantly smaller LV volumes, a difference primarily driven by women, along with lower LVGLS and RVGLS (p < 0.001). In contrast, LA volumes were smaller in the Northeast region. No significant associations were observed between self-reported race and echocardiographic parameters, except for RVGLS and LASr, which were lower in White participants.

**Conclusion:**

This study establishes echocardiographic reference values in a nationally representative Brazilian cohort, confirms expected sex-related differences, and identifies previously unrecognized regional variations in cardiac chamber dimensions. These findings underscore the need for population-specific standards to ensure accurate cardiac quantification.

## Introduction

Echocardiographic assessment of cardiac chamber size, left ventricular (LV) mass, and systolic function is essential for cardiovascular risk characterization.^1 , 2^Therapeutic decisions are frequently guided by parameters of systolic function, which provide valuable information for diagnosis, treatment, and prognosis across a wide range of conditions. In addition, accurate determination of cardiac volumes is critical for the follow-up of patients with heart failure, valvular disease, and cardiac remodeling.^3^

Reference values for normal cardiac measurements may reflect the anthropometric, genetic, socioeconomic, and racial characteristics of specific populations. Brazil, a country of continental dimensions, exhibits marked economic, racial, and cultural diversity. According to the 2022 Census, conducted by Brazil’s Institute of Geography and Statistics, the Brazilian population is predominantly composed of mixed-race individuals (45.31%), followed by White (43.5%), Black (10.2%), Indigenous (0.8%), and Asian (0.4%) populations. In addition, the five major geographic regions show substantial disparities, each with distinct characteristics that may influence health outcomes and phenotypic expression.

Most studies on echocardiographic reference values are based on European^1^or North American populations,^2^which may not be fully representative of the Brazilian population. A recent large-scale, multi-racial study including participants from centers worldwide^3^demonstrated differences in LV diameters and volumes among countries. For example, Brazilian women exhibited higher LV ejection fraction (EF) (LVEF) values compared with those reported in American, European, and Asian cohorts.^3^

In contrast, most studies conducted in Brazil have been limited to smaller, single-center cohorts,^4 , 5^which may not adequately capture the country’s diversity. Therefore, the objective of this cross-sectional study was to establish reference values for echocardiographic parameters in a robust cohort of individuals from the five major Brazilian regions, and to compare findings according to sex and race across regions.

## Methods

### Participants and study setting

Healthy volunteers aged > 18 years, of both sexes, were prospectively enrolled. Participants were recruited from hospital staff, relatives of staff members, and individuals undergoing echocardiographic examinations for health or insurance checkups.

Exclusion criteria included any known cardiovascular or systemic condition that could influence cardiac measurements, including coronary heart disease, diabetes, hypertension, arrhythmias, congenital heart disease, liver or lung disease, thyroid or kidney disorders, and rheumatic diseases. Additional exclusion criteria were the use of cardiovascular medications, body mass index (BMI) > 30 kg/m^2^, athletes (defined as professional athletes or individuals engaging in regular sports activity for > 5 hours/week), pregnancy, suboptimal echocardiographic windows, significant valvular disease (regurgitation or stenosis greater than mild, detected during echocardiography), and LVEF < 50%.

Self-reported race was classified as Mixed-race, White, Black, Asian, or Indigenous. Participants were recruited from 20 echocardiography laboratories across the five major Brazilian regions: nine centers in the Southeast, four in the South, three in the Northeast, two in the Center-West, and two in the North ( Central Illustration ).

### Echocardiographic Measurements

Standard transthoracic echocardiography was performed using commercially available equipment with harmonic imaging capability. Patients were continuously monitored with electrocardiography (ECG) while in the left lateral decubitus position. Gain and depth settings were adjusted to optimize image quality.

2D images were acquired from parasternal long- and short-axis views, as well as apical four-, three-, and two-chamber views. LV end-diastolic (ED) and end-systolic (ES) diameters were measured from the parasternal long-axis view to calculate LV mass, along with septal wall thickness and posterior wall thickness at ED, according to American Society of Echocardiography (ASE) guidelines.

Apical four- and two-chamber views were used to calculate LV ED and ES volumes, stroke volume (SV), and EF using the modified biplane Simpson’s method. Images from apical four-, three-, and two-chamber views with frame rates > 50 frames per second were used for LV global longitudinal strain (LVGLS) analysis.

Images from the right ventricular (RV)-focused apical four-chamber view were obtained for RVGLS and free wall GLS (FWGLS). Left atrial (LA) volumes were measured from apical two- and four-chamber views using Simpson’s method, and LA strain reservoir (LASr) was derived from the standard apical four-chamber view. Strain values were analyzed based on their absolute magnitude, regardless of negative representation. Linear measurement of the LA anteroposterior diameter from the parasternal long-axis view was also performed according to ASE guidelines.^6^

LV diameters and mass, as well as LV and LA volumes, were indexed to body surface area (BSA) using the Du Bois formula.^7^Color Doppler was used to assess all cardiac valves. Continuous- and pulsed-wave Doppler were obtained from pulmonary, tricuspid, mitral, and aortic valves from parasternal and apical views to evaluate intracardiac flow.

Images were stored in digital imaging and communications in medicine (DICOM) format on DVDs and subsequently uploaded to a secure web-based platform for research data storage and management, Research Electronic Data Capture,^8^together with clinical information for offline analysis.

### Analysis of measurements

All image analyses and measurements were performed offline using a vendor-neutral platform capable of storing, viewing, and analyzing echocardiographic images. Strain analysis was conducted offline using AutoStrain^®^software after confirming the location of the ED start and ED end markers (Ultrasound Workspace, Philips-Tomtec).

Image analyses were performed by four researchers from core echocardiography laboratories in São Paulo, Rio de Janeiro, and Bahia.

### Ethical considerations

The study was approved by the Ethics Committee of the coordinating hospital (protocol number 17275419.3.1001.0071) and by the ethics committees of all participating centers. All participants provided written informed consent prior to enrollment. The study was conducted in accordance with the ethical principles of the Declaration of Helsinki and Brazilian National Health Council Resolution No. 466/2012.

### Statistics analysis

Measurements were expressed as mean and standard deviation (SD) for normally distributed variables, or as median and interquartile range for variables with skewed distribution. Continuous variables were also presented as mean ± SD. The distribution of quantitative variables was assessed using the Shapiro-Wilk test. Categorical variables were expressed as percentages.

Group comparisons were performed using the unpaired Student’s *t* test or Mann-Whitney *U* test for continuous variables, and the chi-square test for categorical variables. Analysis of variance (ANOVA) or Kruskal-Wallis tests were used to compare differences among subgroups. Duncan’s test was applied as a post hoc analysis following ANOVA, and the Benjamini-Hochberg procedure was used after Kruskal-Wallis tests. Statistical significance was defined as p < 0.05.

Intraobserver and interobserver variability were assessed using the intraclass correlation coefficient in 10 patients and Bland-Altman plots performed 30 days after the initial measurements ( Figure 1 ; Figure 2 ). Data analysis was conducted using R software, version 4.1.1.

**Figure 1:** – Bland-Altman plots for intraobserver variability of strain measurements. SD: standard deviation.

**Figure 2:** – Bland-Altman plots for interobserver variability in deformation parameters. SD: standard deviation.

## Results

### Sample characteristics

Data collection began in 2021, after the decline of the COVID-19 pandemic. A total of 593 individuals were initially enrolled. A total of 97 participants were excluded for the following reasons: inadequate image storage (n = 75), missing ECG recordings (n = 10), BMI > 30 kg/m^2^(n = 5), absence of informed consent (n = 4), abnormal echocardiographic findings (n = 2; one case of mitral valve prolapse and one of LV systolic dysfunction), and age < 18 years (n = 1). Thus, 496 participants were included in the final analysis.

Participants were recruited from 20 echocardiography laboratories across the five major Brazilian regions; two centers contributed only as reading sites. Most participants (n = 234) were from the Southeast region, the most populous region in Brazil. The remaining sample included 124 individuals from the Center-West, 48 from the Northeast, 45 from the South, and 45 from the North ( Central Illustration ).

Analysis of self-reported race showed that most participants identified as White (56%), followed by Mixed-race (31%) and Black (11%) individuals. A smaller proportion identified as Asian (2%), and no participants reported Indigenous ancestry. The distribution of self-reported race varied significantly by region. Mixed-race individuals accounted for 63% and 53% of participants in the Northeast and North, respectively, whereas in the South, 84% identified as White. Table 1 presents the distribution of self-reported race across regions.

**Table 1:** – Racial characteristics of the Brazilian population according to region

Race	All (n = 496)	SE (n = 234)	CW (n = 124)	NE (n = 48)	S (n = 45)	N (n = 45)
White, n (%)	277 (56.3)	130 (55.6)	76 (61.3)	14 (29.2)	38 (84.4)	19 (42.2)
Mixed, n (%)	157 (31.3)	61 (26.0)	35 (28.2)	30 (62.5)	7 (15.6)	24 (53.3)
Black, n (%)	53 (10.6)	34 (14.5)	13 (10.5)	4 (8.3)	0 (0)	2 (4.4)
Asian, n (%)	9 (1.8)	9 (3.9)	0 (0)	0 (0)	0 (0)	0 (0)

CW: Center-West; N: North; NE: Northeast; S: South; SE: Southeast.

The study population included a slightly higher proportion of women (55%). The mean age was 41 ± 15 years (range: 18-100 years), with no significant difference between men and women (p = 0.30). As expected, women had significantly lower anthropometric measures than men, including weight, height, BSA, and BMI. Women also had higher resting heart rates (p < 0.001). A detailed summary of demographic characteristics for the overall cohort and stratified by sex is presented in Table 2 .

**Table 2:** – Demographics and heart rate of all individuals (men and women)

Variable	All (n = 496)	Men (n = 224)	Women (n = 272)	p-value
**Age (years)**				0.312
Mean [SD] (95% CI)	41.4 [15.0] (40.0-42.7)	40.5 [14.7] (38.6-42.4)	42.0 [15.2] (40.2-43.9)	
Median (95% CI)	39 (37-41)	38 (35-40)	40 (38-43)	
**Weight (kg)**				< 0.001
Mean [SD] (95% CI)	71.0 [11.5] (70.0-72.0)	77.9 [10.0] (76.6-79.2)	65.3 [9.4] (64.2-66.5)	
Median (95% CI)	70 (70-72)	77 (72-84)	64 (63-66)	
**Height (cm)**				< 0.001
Mean [SD] (95% CI)	167.7 [9.2] (166.9-168.5)	174.3 [7.3] (173.3-175.2)	162.3 [6.7] (161.5-163.1)	
Median (95% CI)	168 (166-169)	174 (172-175)	162 (161-164)	
**BSA (m2)**				< 0.001
Mean [SD] (95% CI)	1.80 [0.18] (1.78-1.82)	1.93 [0.15] (1.91-1.95)	1.70 [0.14] (1.68-1.71)	
Median (95% CI)	1.79 (1.70-1.82)	1.91 (1.89-1.93)	1.69 (1.67-1.71)	
**BMI (kg/m2)**				< 0.001
Mean [SD] (95% CI)	25.1 [2.8] (24.9-25.4)	25.6 [2.5] (25.3-25.9)	24.7 [2.9] (24.4-25.1)	
Median (95% CI)	25.2 (24.8-25.5)	25.6 (25.3-25.9)	24.5 (24.0-25.0)	
**Heart rate (bpm)**				< 0.001
Mean [SD] (95% CI)	69.3 [10.4] (68.5-70.3)	67.3 [10.2] (66.0-68.7)	71.1 [10.3] (69.8-72.3)	
Median (95% CI)	69 (68-70)	66 (60-74)	71 (70-73)	

BMI: body mass index; BSA: body surface area; SD: standard deviation.

### Echocardiographic parameters

All LV dimensions, volumes, and LV mass were larger in men, and these sex-related differences remained significant after indexing for BSA. Both LA anteroposterior diameter and LA volume were greater in men, although indexed LA volumes were similar between sexes.

Regarding LV systolic function, women exhibited slightly higher LVEF values (63.1 ± 4.6% vs. 61.4 ± 4.3% for women and men, respectively; Supplementary Table 3). Most strain parameters demonstrated marked sex-related differences. Women had significantly higher LVGLS, RVGLS, and RV FWGLS compared with men. Although LASr also tended to be higher in women, the difference did not reach statistical significance (p = 0.089).

### Regional differences in demographics and left ventricular parameters

Sex distribution was similar across all Brazilian regions. However, individuals from the Northeast were slightly younger than those from the Southeast and Center-West (p = 0.003). BSA was slightly higher in the Southeast compared with the South (p = 0.027).

Significant regional variation was observed in LV measurements. LV ED volumes (p < 0.001), ES volumes (p = 0.017), and diameters (p < 0.05) were smaller in the Center-West, particularly compared with the North, even after indexing for BSA. Stroke volume was also lower in the Center-West (p < 0.001), and this difference remained significant after indexing (p < 0.001). In contrast, LVEF did not differ significantly among regions.

LV mass, LV mass index, and septal wall thickness were similar across regions; however, posterior wall thickness was significantly lower in the Northeast (7.7 ± 0.9 mm) compared with the North (8.2 ± 1.1 mm; p = 0.006).

Regional variation was also observed in myocardial deformation parameters. LVGLS differed significantly among regions (p < 0.001), with the Center-West consistently showing the lowest values in post hoc comparisons. A similar pattern was observed for RVGLS and RV FWGLS (both p < 0.001), again with the lowest values in the Center-West. In contrast, LASr also varied significantly by region (p = 0.043), with the highest values observed in the Northeast (Supplementary Table 4).

### Sex-Related differences across regions

Within regions, men showed minimal differences in echocardiographic parameters; LV ED volume and stroke volume were lower in the Center-West. Among women, however, significant regional differences were observed in both demographic and echocardiographic variables.

Women from the Northeast were younger than those from other regions. Echocardiographically, the Center-West showed the smallest LV ED and ES diameters (p < 0.05) as well as the smallest LV ED and ES volumes (p < 0.001), whereas the North had the largest volumes.

LA volume (p = 0.002) and indexed LA volume (p = 0.003) were lower in women from the Northeast. Although LV mass and mass index did not differ significantly among women, some regional variation was observed in myocardial thickness. Specifically, posterior wall thickness (p = 0.006) and septal wall thickness (p = 0.005) were significantly lower in women from the North, a pattern observed only in the female subgroup.

For strain parameters, regional differences were observed in both sexes. Among men, LVGLS and RVGLS varied significantly across regions, with the lowest LVGLS observed in the Center-West (19.3 ± 1.2%) and the highest in the South (21.8 ± 2.3%). RVGLS was also lower in the Center-West compared with other regions.

Among women, LVGLS, RVGLS, RV FWGLS, and LASr all showed significant regional differences. Similar to men, the Center-West had the lowest LVGLS (20.4 ± 1.8%) and RVGLS (21.8 ± 2.2%) in women. Notably, women from the South had the highest LVGLS (23.3 ± 2.3%) and LASr (43.7 ± 5.5%). Post hoc analyses confirmed that the Center-West consistently differed from multiple other regions in both sexes, particularly for LV and RV strain (Supplementary Table 5).

### Differences according to self-reported race

Because race has been associated with variation in echocardiographic measurements,^4^we assessed potential differences between the two largest racial subgroups: Mixed-race (n = 157) and White individuals (n = 277). Indexed LV ED diameter was significantly greater in White participants. In contrast, RVGLS (23.1 ± 2.8% vs. 22.5 ± 2.5%; p = 0.036) and LASr (41.5 ± 5.8% vs. 40.3 ± 5.9%; p = 0.039) were significantly higher in the Mixed-race group.

No significant differences were found for LVGLS or RV FWGLS. Among demographic variables, only age differed significantly, with White participants being slightly older (p = 0.025); all other characteristics were comparable between groups (Supplementary Table 6).

## Discussion

### Population

This is the first prospective, multicenter study to provide 2D echocardiographic and myocardial deformation reference parameters for the Brazilian adult population, including participants from all five major regions of the country, with analyses stratified by sex and self-reported race. According to the 2022 Census,^5^most of the Brazilian population is composed of individuals of mixed race. However, in our sample, most participants self-identified as White (57%), followed by Mixed-race (31%) and Black (10%), with a small proportion identifying as Asian (2%).

We recognize that many individuals who self-report as White may have characteristics consistent with mixed ancestry. The Brazilian population reflects a complex admixture of ancestries, primarily Portuguese, African, and Indigenous, later supplemented by European and Japanese migration, resulting in a highly heterogeneous genealogical background. As such, individuals may have multiple ancestral origins, although this may not always be reflected in self-reported race.

Notably, a greater proportion of participants were recruited from private health care facilities rather than public institutions. Because Mixed-race and Black individuals in Brazil generally have less access to private health care than White individuals, this recruitment pattern may have introduced selection bias.^6^Consistent with the most recent census data, the South region had a higher proportion of White individuals, whereas the Northeast and North regions were predominantly composed of Mixed-race individuals.

Regarding sex distribution, our study included a slightly higher proportion of women (55%), closely reflecting the Brazilian population, in which women account for approximately 51%. Most participants were recruited from the Southeast region, which contains the country’s largest and most populous cities and represents more than 40% of the Brazilian population, highlighting the country’s marked geographic disparities.

We included individuals across a wide age range (18-100 years), with a mean age of 41 ± 15 years, which is slightly higher than the national average age reported in the most recent census (35 years). Because of these characteristics, we believe our sample may be broadly representative of the Brazilian population. However, compared with some international studies, our cohort is younger, with a mean age of 45.8 years in the Normal Reference Ranges for Echocardiography study^7^and 57 years in a longitudinal Norwegian cohort.^8^

### Sex differences

In our cohort, LV chamber dimensions were larger in men, consistent with previous reports, and these differences persisted even after indexing for BSA. Mean values were lower than those reported in other studies,^8^which likely reflects demographic differences, including smaller height, weight, and BSA, which are known to influence cardiac structure.

LA volumes were also significantly greater in men; however, these differences were no longer significant after indexing for BSA. Notably, even after indexation, LA volumes in our population remained lower than those reported in European cohorts (25.9 ± 6.3 mL/m^2^in men and 25.6 ± 6.0 mL/m^2^in women).

Regarding LVEF, our analysis demonstrated higher values in women compared with men, in agreement with previous studies and potentially related to sex-related differences in stroke volume or afterload.^9^Our findings are consistent with those of the World Alliance Societies of Echocardiography study, although the Brazilian cohort in that study was derived from a single center. Earlier studies conducted in Brazil also proposed reference values for the population,^10 , 11^but these were likewise single-center investigations involving individuals from only one region.

Technological advances in imaging and improved spatial resolution have enhanced visualization of cardiac structures, enabling more accurate two-dimensional measurements. With respect to myocardial deformation, our findings confirm that strain parameters are influenced by sex, with women consistently exhibiting higher (more negative) strain values in both ventricles, supporting previously reported sex-related differences.^12 - 14^

In the Brazilian Longitudinal Study of Adult Health (ELSA-Brasil),^15^lower LVGLS values were reported, although higher values in women were also confirmed. Unlike the present study, echocardiographic images in ELSA-Brasil were not prospectively acquired specifically for strain analysis, and LV strain was derived only from apical two- and four-chamber views, without inclusion of the three-chamber view. This methodological difference may explain the lower values observed.

The more negative LVGLS observed in women compared to men is in line with earlier studies suggesting that women exhibit more efficient systolic mechanics, potentially due to differences in myocardial fiber orientation, lower afterload, and hormonal influences. Additionally, RVGLS and RV FWGLS were significantly higher in women, likely reflecting sex differences in RV geometry, ventricular-vascular coupling and hormonal effects. Elevated estradiol in women on hormone therapy correlates with improved RVEF and decreased ES volume, supporting estrogen’s beneficial influence on RV structure and function.^16^

Importantly, RV strain has emerged as a more sensitive marker of RV function than traditional parameters such as tricuspid annular plane systolic excursion,^17^and sex-specific normal values should be considered to avoid under- or overestimation of RV dysfunction. Experimental studies have also shown that strain changes allow detection of sex-specific cardiac alterations at earlier stages compared with conventional echocardiographic methods.^18^

These findings underscore the importance of sex stratification in echocardiographic normative datasets, particularly for myocardial deformation parameters, which are increasingly incorporated into clinical guidelines for cardiac function assessment. In addition to being more sensitive and reproducible than traditional measures such as EF,^19^GLS demonstrates lower inter- and intraobserver variability, especially when measured using automated software such as AutoStrain.^20^

### Major geographical regions

Although the study aimed to achieve representative sampling across Brazil’s five regions, most participants were recruited from the Southeast, the most densely populated area of the country. Nevertheless, our analysis identified small but statistically significant regional differences. LV diameters and volumes were smaller in the Center-West region. This finding may be partially related to the racial composition of this population, which includes greater Indigenous ancestry, although less prominently than in the North region.

For LA volumes, women from the Northeast showed smaller values, particularly compared with those from the South, even after adjustment for BSA. This difference may partly reflect the younger age of participants from the Northeast, as LA volume tends to increase with age due to progressive diastolic dysfunction and atrial remodeling. Given the prognostic relevance of LA volumes, these regional variations warrant consideration.^21^Septal and posterior wall thicknesses were also lower in women from the Northeast, likely reflecting a combination of genetic and racial influences.

Regarding myocardial deformation, significant regional variation was observed in LVGLS, RVGLS, RV FWGLS, and LASr. The Center-West consistently showed lower deformation values across all parameters, possibly related to smaller LV dimensions and consequently lower volume demands. In contrast, participants from the Northeast exhibited higher strain values, particularly for LVGLS and RV FWGLS, which may be associated with differences in anthropometry, physical activity, or genetic background. Higher LASr values in the Northeast may also reflect better atrial compliance related to the younger age of this group.

Overall, these findings demonstrate notable geographic variability in myocardial deformation parameters, particularly among individuals from the Center-West, who consistently showed lower LV and RV strain values in both sexes. The more pronounced differences observed in women, including significant variation in RV FWGLS and LASr, underscore the importance of establishing sex- and region-specific reference values for accurate interpretation of strain parameters in diverse populations.

### Self-Reported race

For any given population, normal reference values are influenced by anthropometric and racial characteristics.^7^Evidence suggests that racial and racial differences may also affect morbidity and mortality, particularly in the presence of socioeconomic disparities.^22^Reference ranges in echocardiography vary according to race,^4^which may contribute to differences in LV and LA size.

In our cohort, subtle race-related differences in subclinical myocardial function were observed. Mixed-race individuals showed greater RVGLS and higher LASr. These variations may reflect physiological adaptations related to genetic, hemodynamic, or anthropometric factors. Although such differences could partly be associated with age-related cardiac remodeling, their clinical relevance appears limited, as all values remained within normal ranges.

Cardiac dimensions are also closely related to body size, and in this study, BSA was comparable across groups. A previous Brazilian study of reference values found no association between echocardiographic parameters and race.^10^Finally, similar to the Brazilian Census, race in this study was based on self-declaration. Racial identification in Brazil is largely based on phenotype rather than familial lineage; therefore, the use of racially diverse cohorts is essential to minimize potential misclassification in echocardiographic interpretation.

### Study limitations

Despite the large sample size, some regions included relatively small numbers of participants, limiting the ability to fully assess regional differences. Additionally, the number of Asian participants was small, preventing meaningful comparisons among racial subgroups. This study did not include Indigenous individuals, who represent approximately 0.83% of the Brazilian population (nearly 2 million people). Research involving Indigenous communities in Brazil requires specific authorization from CONEP and often presents logistical challenges related to limited accessibility. Therefore, our findings cannot be generalized to this group.

Although multiple ultrasound platforms were used across participating sites, all acquisitions were exported in DICOM format and analyzed using vendor-independent software, reducing intersystem variability. Finally, age-based subgroup analyses were not performed, as only 52 participants were older than 65 years, and it was difficult to identify individuals in this age group who met all inclusion criteria (absence of hypertension, diabetes, or other cardiovascular diseases).

## Conclusion

This is the first multicenter study to provide nationally representative echocardiographic reference values for Brazil. In addition to confirming sex-related differences, we identified previously unrecognized regional variability, highlighting the importance of population-specific echocardiographic standards.

## Supplemental Materials

Table 3
