# Supplementary material for: Errata
Source: Arq Bras Cardiol. 2026 May 8;123(3):e20260248. [Article in Portuguese] doi: 10.36660/abc.20260248 (PMC13398894; doi:10.36660/abc.20260248)
Supplement: Translation

# Translation: Erratum

**DOI:** 10.36660/abc.20260248i

February 2026 Issue, vol. 123(2):e20250628i

In the Original Article “Reference Echocardiographic Values for Cardiac Chambers in Brazil: A Multiregional, Multi–Racial Study”, with DOI number: https://doi.org/10.36660/abc.20250628i, published in the journal Arquivos Brasileiros de Cardiologia, Arq Bras Cardiol. 2026; 123(2):e20250628, on page 1, include the institution: Complexo Hospitalar Américas, 27, Rio de Janeiro, RJ – Brazil. Change the numbering of Dr. Alex dos Santos Felix’s affiliations from 15,20 to 14,20,27.
